# Antibiotics in the clinical pipeline as of December 2022

**DOI:** 10.1038/s41429-023-00629-8

**Published:** 2023-06-08

**Authors:** Mark S. Butler, Ian R. Henderson, Robert J. Capon, Mark A. T. Blaskovich

**Affiliations:** Centre for Superbug Solutions, Institute for Molecular Bioscience, The University of Queensland, St Lucia, Brisbane, 4072 Australia

**Keywords:** Drug discovery, Microbiology

## Abstract

The need for new antibacterial drugs to treat the increasing global prevalence of drug-resistant bacterial infections has clearly attracted global attention, with a range of existing and upcoming funding, policy, and legislative initiatives designed to revive antibacterial R&D. It is essential to assess whether these programs are having any real-world impact and this review continues our systematic analyses that began in 2011. Direct-acting antibacterials (47), non-traditional small molecule antibacterials (5), and β-lactam/β-lactamase inhibitor combinations (10) under clinical development as of December 2022 are described, as are the three antibacterial drugs launched since 2020. Encouragingly, the increased number of early-stage clinical candidates observed in the 2019 review increased in 2022, although the number of first-time drug approvals from 2020 to 2022 was disappointingly low. It will be critical to monitor how many Phase-I and -II candidates move into Phase-III and beyond in the next few years. There was also an enhanced presence of novel antibacterial pharmacophores in early-stage trials, and at least 18 of the 26 phase-I candidates were targeted to treat Gram-negative bacteria infections. Despite the promising early-stage antibacterial pipeline, it is essential to maintain funding for antibacterial R&D and to ensure that plans to address late-stage pipeline issues succeed.

## Introduction

Antibiotics are the foundation of modern medicine but are becoming increasingly ineffective due to growing levels of antimicrobial resistance, threatening global health. The adverse impact of drug-resistant infections is highlighted by a seminal analysis of the global burden of bacterial antimicrobial resistance in 2019, with 1.27 million deaths directly attributed to, and 4.9 million deaths associated with, resistant bacteria [1]. The development of new antibiotics, particularly new chemotypes or classes that can overcome existing resistance mechanisms, has been hindered by a failure of the healthcare system marketplace to adequately recognize and compensate for these products [2–4]. In addition to improved generic antibiotic sales, branded antibiotic prices have fallen since 2001 [5], aggravating the economic challenges. Recognition of the antibiotic crisis has led to the establishment of targeted funding initiatives for antibiotic development such as the Combating Antibiotic-Resistant Bacteria Biopharmaceutical Accelerator (CARB-X) [6], INCATE [7], REPAIR Impact Fund [8], and the AMR Action Fund [9, 10], testing of new incentives to reimburse pharmaceutical companies such as a subscription ‘Netflix’ model [11–14], and legislative initiatives such as the PASTEUR (The Pioneering Antimicrobial Subscriptions To End Up surging Resistance) Act in the United States [15, 16]. There has also been an increase in the number of “non-traditional” antibacterials [17–21] being actively evaluated in clinical trials [21, 22]. Non-traditional antibacterials can be small molecules, monoclonal antibodies (mAbs), proteins or live biotherapeutics such as bacteria and bacteriophages that primarily affect bacteria growth or virulence indirectly with varying mechanisms such as toxin binding, cell adherence reduction, inhibition of antivirulence targets and drug resistance modification [21].

To assess whether these activities are improving the status quo, we have monitored antibacterial drug development since 2011 with reviews published in 2019 [23], 2015 [24], 2013 [25] and 2011 [26]. Complementary reviews with different approaches and analyses (but often with few or no chemical structures) are available. The Pew Trusts developed an online pipeline tracker that allows the visualization of changes in the pipeline from 2014–2020 [27], but their antibiotic resistance project was discontinued in December 2021 [28]. In 2022, the WHO published a report on antibacterial agents in both preclinical and clinical development in 2021 [22] and a journal article in 2022 [21]. The WHO also recently reviewed the preclinical and clinical antibacterial vaccine pipeline [29]. A 2021 review critically analyzed why compounds with Gram-negative (G-ve) activity have fallen out of the pipeline over the past decade [30], while two 2020 reviews covered both the clinical [31] and preclinical [32] antibacterial pipelines, with a third providing an overview of ‘novel’ antibacterial agents in various stages of development [33]. Reviews of patents from 2010–2021 focusing on compounds with activity against multi-drug resistant (MDR) G-ve bacteria [34], antibacterial combinations [35], and discovery strategies [36] have also been published.

**Fig. 1:**
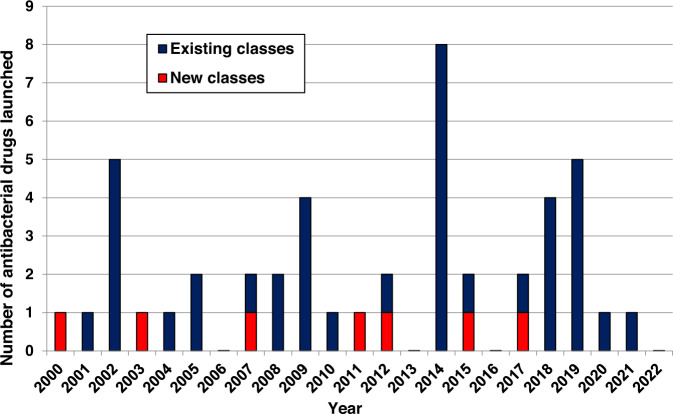
New small molecule antibacterial drugs and BL/BLI combinations launched from January 2000 to December 2022 with new classes highlighted

**Fig. 2:**
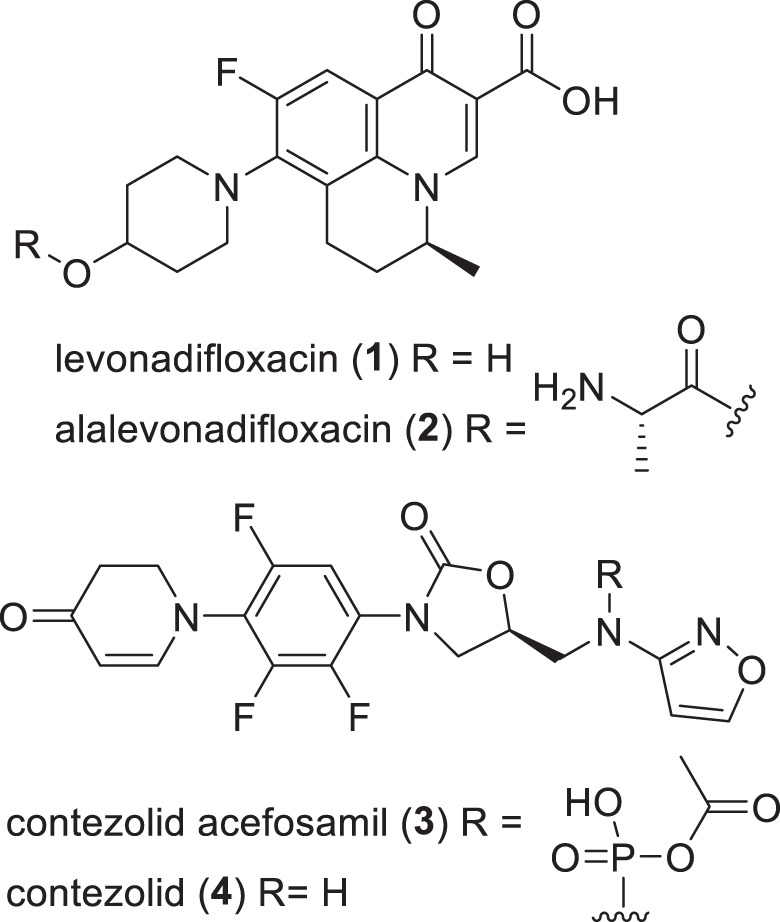
Structures of the recently lauched antibacterial drugs

**Fig. 3:**
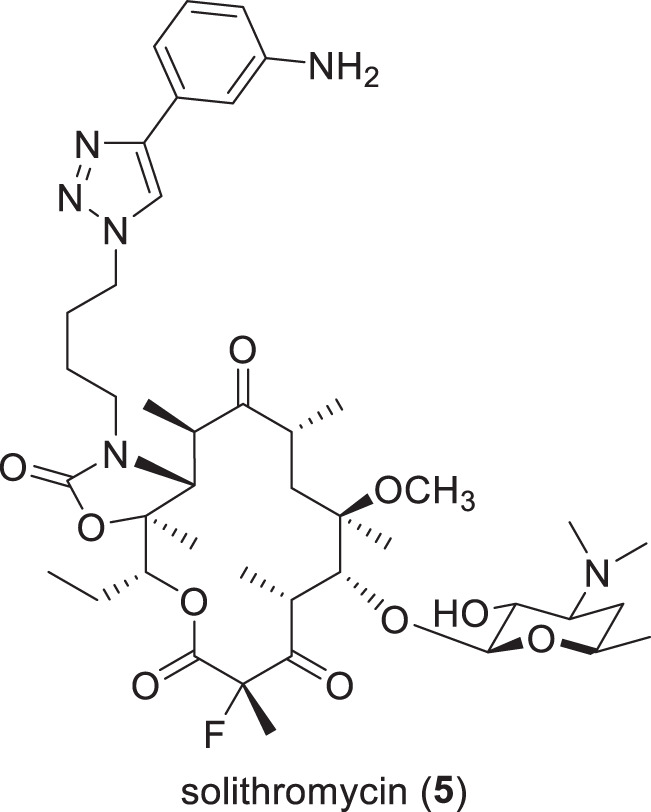
Structure of the antibacterial in the NDA and MAA development stage (Table 3)

**Fig. 4:**
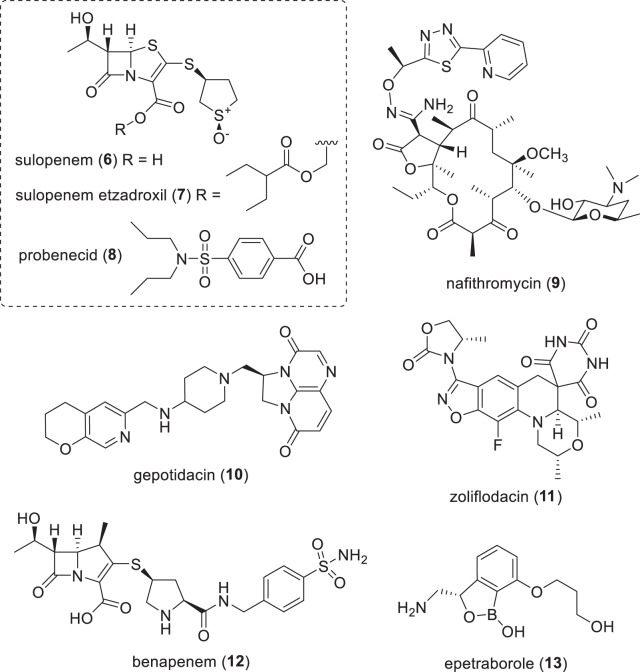
Structures of compounds in phase-III clinical trials (Table 3)

**Fig. 5:**
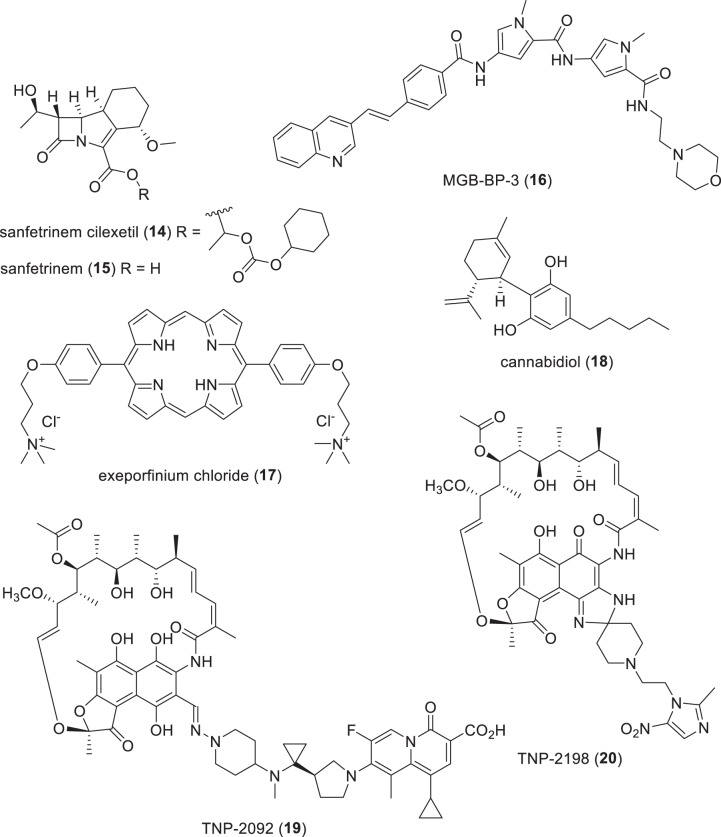
Structures of NP-derived compounds in phase-II clinical trials (Table 4)

**Fig. 6:**
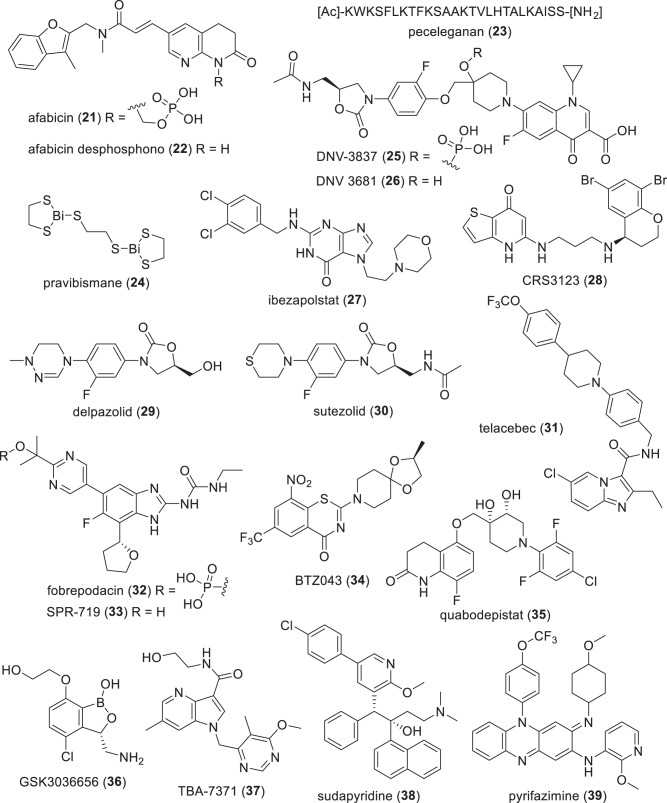
Structures of synthetic compounds in phase-II clinical trials (Table 4)

**Fig. 7:**
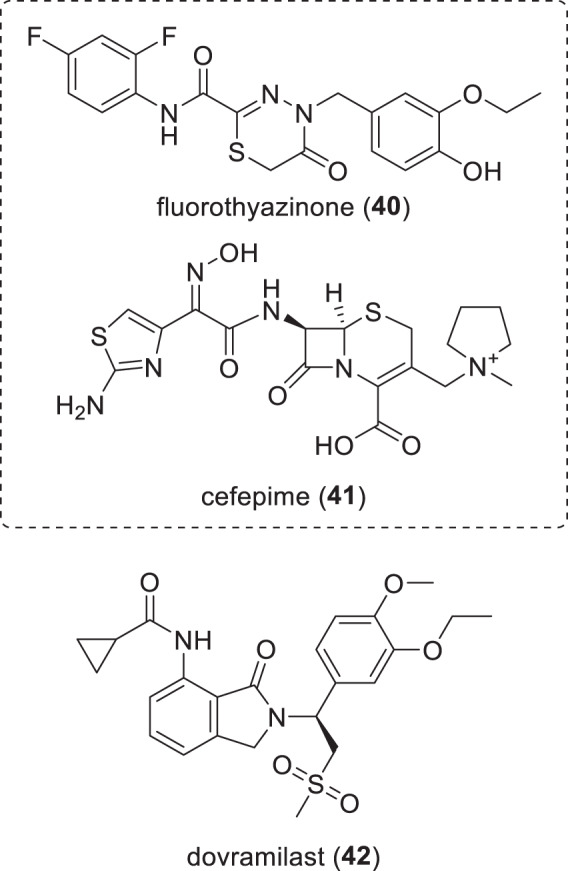
Structures of small molecule non-traditional antibacterials in phase-II clinical trials (Table 4)

**Fig. 8:**
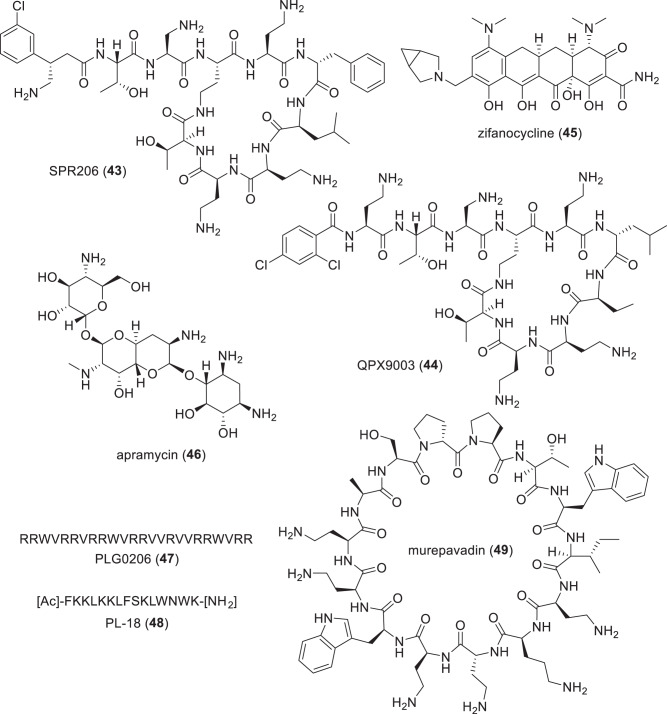
Structures of NP and peptide-derived compounds in phase-I clinical trials (Table 5)

**Fig. 9:**
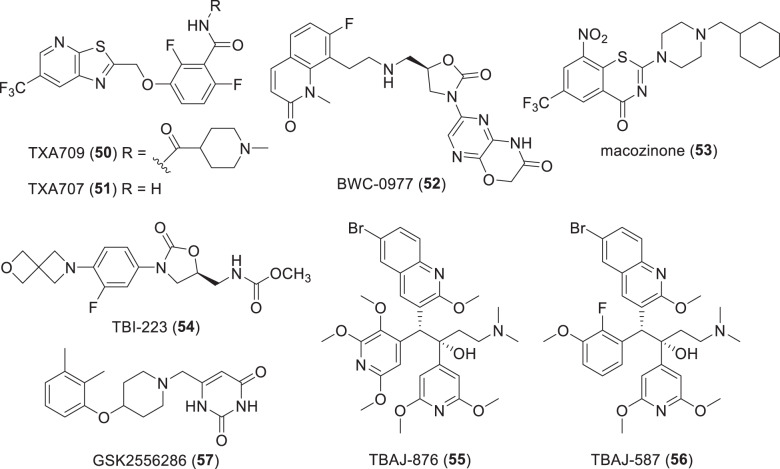
Structures of synthetic-derived compounds in phase-I clinical trials (Table 5)

**Fig. 10:**
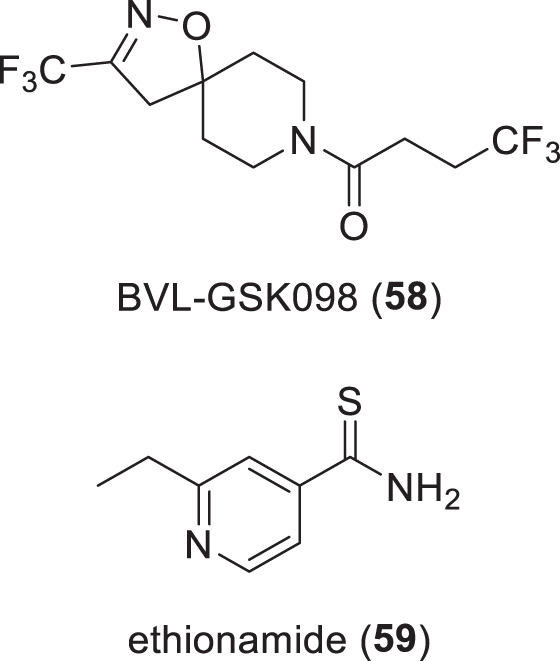
Structures of publicly disclosed small molecule non-traditional antibacterials in phase-I clinical trials (Table 5)

**Fig. 11:**
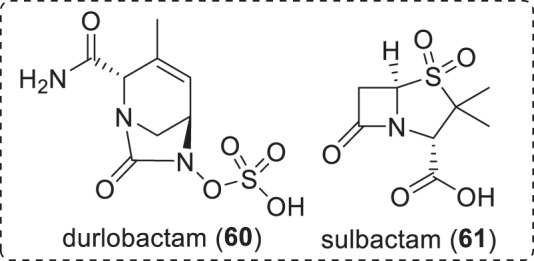
Structures of BLI and associated β-lactam antibacterial in NDA/MAA filing (Table 6)

**Fig. 12:**
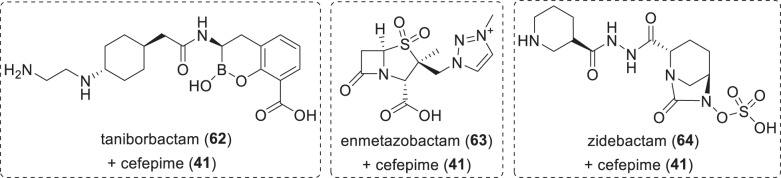
Structures of BLIs in phase-III clinical trials (Table 6)

**Fig. 13:**
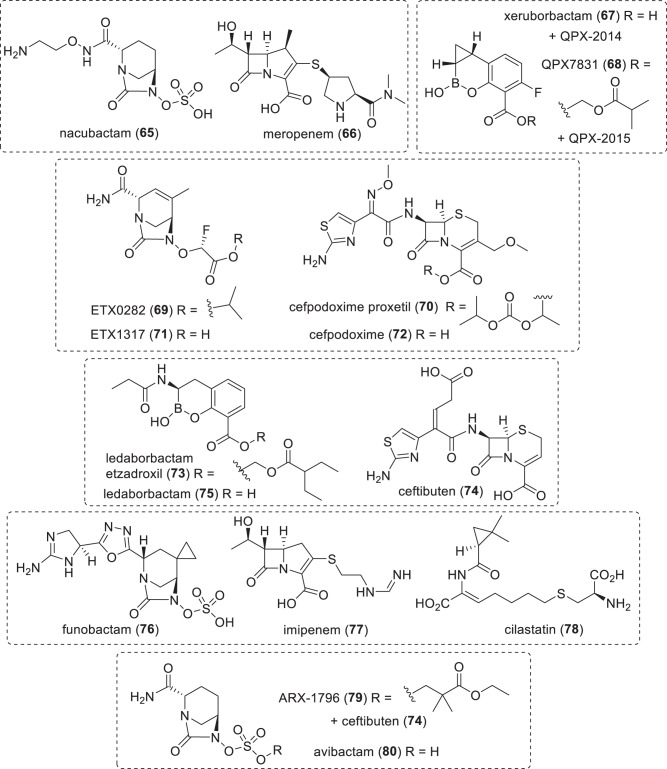
Structures of BLIs and associated β-lactam antibiotics in phase-I clinical trials (Table 6)

**Fig. 14:**
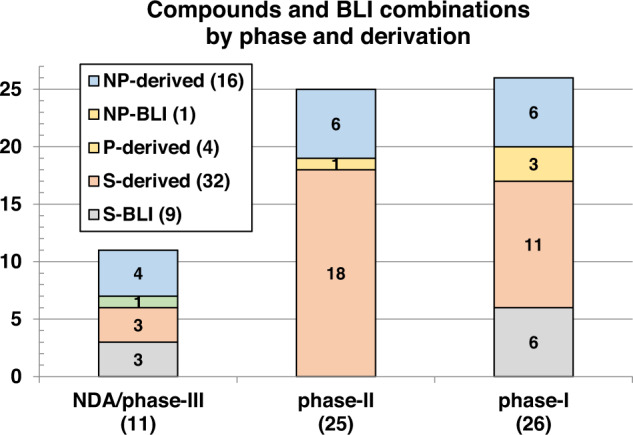
Compounds under clinical evaluation divided into development phases and their lead derivation source: natural product (NP) (NP-derived and NP-BLI), protein/mammalian peptide (P-derived) and synthetic (S) (S-derived and S-BLI)

**Fig. 15:**
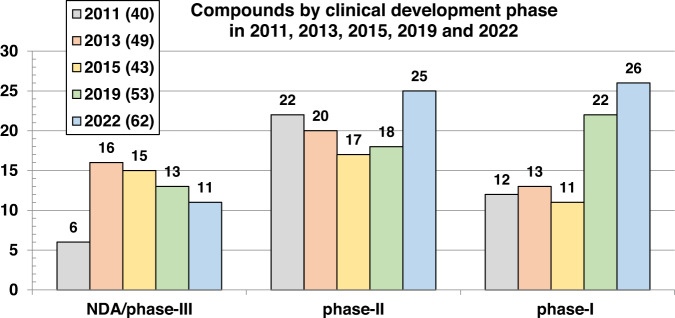
Comparison of the numbers of compounds undergoing clinical development as of 2011 [26], 2013 [25], 2015 [24], 2019 [23] and 2022 by development phase

**Fig. 16:**
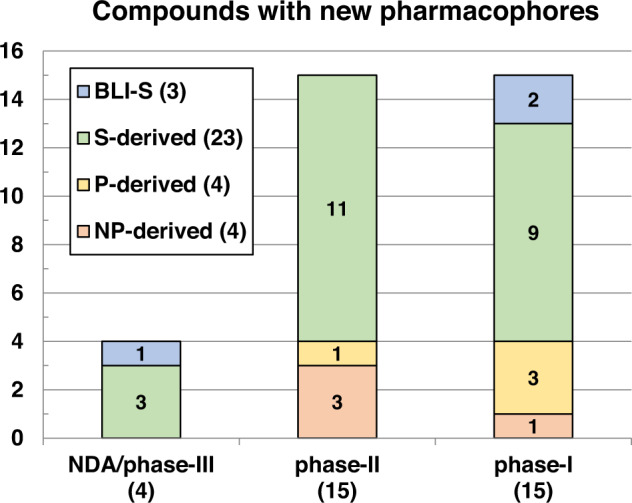
Antibacterial compounds [natural product (NP), synthetic (S), protein/mammalian peptide (P)] and β-lactamase inhibitors (BLI)] with new antibacterial pharmacophores divided into development phases and their lead derivation source

**Fig. 17:**
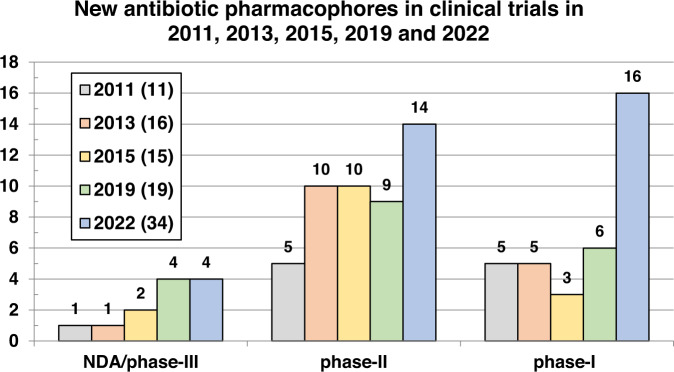
Comparison of the numbers of novel antibacterial pharmacophores undergoing clinical development in 2011 [26], 2013 [25], 2015 [24], 2019 [23] and 2022 by development phase

This review catalogs the small molecule antibacterial drugs launched since January 2012 in Table 1 and the yearly number of first-time antibacterial drugs launched by year since 2000 (Fig. 1, Table S1). International Nonproprietary Names (INN) are used for compound names when available. For completeness, Table 2 lists the non-traditional antibacterial drugs launched during this period. The new antibacterial drugs approved since the previous 2019 review [23], levonadifloxacin (**1**) and its prodrug alalevonadifloxacin (**2**), and contezolid acefosamil (**3**) (Fig. 2), are analyzed. Consistent with previous reviews in this series [23–26], small molecule antibacterials (both traditional and non-traditional) and β-lactamase/β-lactam inhibitor (BL/BLI) combinations that are being evaluated in phase-I, -II, or -III clinical trials and under pre-approval regulatory evaluation as of 31 December 2022 are summarized (Tables 3–6, Figs. 3–13), along with their development status, mode of action (MoA), spectra of activity, historical discovery, and lead compound origin (natural product (NP), synthetic (S) or protein/mammalian peptides (P)). In the previous 2019 review [23], one antibody drug conjugate (ADC), DSTA4637S, was discussed, but its development has since been halted (Table 7). The clinical trial study codes, which are predominantly from ClinicalTrials.gov (NCT), are listed in parentheses for each trial, while non-registered trials are referenced at least in a Press Release or peer-reviewed publication. An overview of the drug development and approval process, on-line clinical trial databases antibiotic clinical trial categories and abbreviations can be found in the Supplementary Information. Prodrugs are grouped together with their active metabolites, while ongoing trials of antibacterial drugs already approved anywhere in the world are presented in Table S2. Compounds where no development activity has been identified since 2018 are listed in Table 7. The antibacterials in clinical development have been further analyzed by phase and source derivation (Fig. 14) and also compared with data reported in our 2011 [26], 2013 [25], 2015 [24] and 2019 [23] reviews (Fig. 15). An analysis of new antibacterial pharmacophores (Table 8, Figs. 16 and 17) and administration routes (Figs. S1 and S2) is also included. The administration routes in this review are as follows: po (oral), IV/po (intravenous oral switch); IV (intravenous), IV/topical (IV and topical), po topical (orally administered for *Clostridioides difficile* (formally *Clostridium* [37]) infections (CDI)), oral, topical and inhalation. The ‘po topical’ term distinguishes between oral administration to treat *C. difficile* infections and the gut microbiome compared to topical administration via creams, sprays, and eyedrops.

**Table 1:** Small molecule antibacterial drugs and β-lactamase inhibitor (BLI) combinations launched from January 2013 to December 2022

Year approved	Drug name^a,b^	Class	Country of first approval	Therapeutic indication(s)	Lead source
*Small molecule drugs*					
2014	delamanid	nitroimidazole	Europe	TB	S
2014	dalbavancin	glycopeptide	USA	G+ve SSSI	NP
2014	oritavancin	glycopeptide	USA	G+ve SSSI	NP
2014	tedizolid phosphate (prodrug)	oxazolidinone	USA	G+ve cSSSI	S
2014	nemonoxacin	quinolone	Taiwan	G+ve /G-ve cSSSI	S
2014	morinidazole^c^	nitroimidazole	China	G+ve/G-ve gynecological and suppurative appendicitis	S
2014	finafloxacin^d^	fluoroquinolone	USA	acute otitis externa	S
2015	zabofloxacin	fluoroquinolone	South Korea	G+ve/G-ve CABP	S
2017	delafloxacin	fluoroquinolone	USA	G+ve/G-ve ABSSSI and CABP	S
2018	plazomicin	aminoglycoside	USA	G-ve UTI	NP
2018	eravacycline	tetracycline	Europe	G+ve/G-ve IAI	NP
2018	omadacycline	tetracycline	USA	G+ve/G-ve CABP and ABSSSI	NP
2018	sarecycline^d^	tetracycline	USA	G+ve acne	NP
2019	pretomanid	nitroimidazole	USA	TB	S
2019	lefamulin	pleuromutilin	USA	G+ve/G-ve CABP	NP
2019	lascufloxacin	fluoroquinolone	Japan	G+ve/G-ve CABP and sinusitis	S
2019	cefiderocol	cephalosporin siderophore	USA	G-ve cUTI and bacterial infections	NP
2020	levonadifloxacin (**1**); alalevonadifloxacin (**2**) (prodrug)	fluoroquinolone	India	G+ve/G-ve ABSSSI	S
2021	contezolid acefosamil (**3**) (prodrug)	oxazolidinone	China	G+ve cSSSI	S
*BL/BLI combination drugs*					
2014	Zerbaxa: ceftolozane + tazobactam^e^	BL + BLI	USA	G-ve cUTI, cIAI and HAP/VAP	NP + NP
2015	Avycaz: avibactam (**80**)^b^ + ceftazidime^e^	DBO BLI + BL	USA	G-ve cUTI, cIAI and HAP/VAP	S + NP
2017	Vabomere/Vaborem: vaborbactam^b^ + meropenem^e^ (**66**)	boronate BLI + BL	USA	G-ve cUTI, cIAI and HAP/VAP	S + NP
2019	Recarbrio: relebactam + imipenem (**77**)^e^ + cilastatin (**78**)^e^	DBO BLI + BL+ renal dehydropeptidase inhibitor	USA	G-ve cUTI, cIAI and HAP/VAP	S + NP + S

*ABSSSI* acute bacterial skin and skin structure infections, *BLI* β-lactamase inhibitor, *BL* β-lactam, *CABP* community-acquired bacterial infections, *DBO* diazabicyclooctane, *HAP/VAP* hospital/ventilator-associated pneumonia, *cIAI* complicated intra-abdominal infections, *NP* natural product, *S* synthetic, *SSSI* skin and skin structure infections, *cSSSI* complicated SSSI, *UTI* urinary tract infections, *cUTI* complicated UTI, *TB* tuberculosis, *USA* United States of America

^a^The structures of antibacterial drugs approved from 2010–2022 are in Fig. 2 and those approved from 2000–2019 can be found in previous reviews [23–26]

^b^First member of a new antibacterial or BLI class approved for human therapeutic use

^c^Also approved for the treatment of amoebiasis and trichomoniasis

^d^Approved for topical use

^e^First launches: tazobactam in 1992, ceftazidime in 1983, meropenem (**66**) in 1998, and imipenem (**77**) + cilastatin (**78**) in 1985

**Table 2:** Non-traditional antibacterial drugs launched from January 2013 to December 2022

Year approved	Drug name	Class	Country of first approval	Therapeutic indication(s)	Lead source
2016	obiltoxaximab	mAb	USA	G+ve anthrax	mAb
2016	bezlotoxumab	mAb	USA	G+ve anthrax	mAb
2022	Rebyota (RBX2660)	microbiome	USA	G+ve CDI	human

*CDI* *C. difficile* infection, *G+ve* Gram-positive bacteria, *mAb* monoclonal antibody, *USA* United States of America

**Table 3:** Antibiotics with NDA/MAA submitted or in phase-III clinical trials (structures in Figs. 3 and 4)

Name (synonym)^a^	Compound class (lead source)	Mode of action^a^	Administration; indication (developer)
*NDA/MAA*			
solithromycin (**6**) (T-4288)	erythromycin (NP)	protein synthesis inhibition	IV/po; respiratory tract infection (FUJIFILM Toyama)
*Phase-III*			
sulopenem (**6**) (IV) sulopenem etzadroxil (**7**) (prodrug) + probenecid (**8**)	penem (NP)	PBP (cell wall)	po; uUTI, cUTI and cIAI (Iterum Therapeutics)
nafithromycin (**9**) (WCK 4873)	macrolide (NP)	protein synthesis	po; CABP (Wockhardt)
gepotidacin (**10**) (GSK-2140944)	triazaacenaphthylene (S)	DNA gyrase (GyrA) — different to quinolones	po; UTI and gonorrhea (GSK)
zoliflodacin (**11**) (ETX0914)	spiropyrimidinetrione (S)	DNA gyrase (GyrB)	po; gonorrhea (Innoviva / GARDP)
*Phase-II/III*			
benapenem (**12**)	carbapenem (NP)	PBP (cell wall)	IV; UTI (Sihuan Pharmaceuticals)
epetraborole (**13**) (BRII-658)	oxaborole (S)	leucyl-tRNA synthetase (LeuRS) – protein synthesis	po; NTM with a focus on *M. avium* (AN2 Therapeutics / Brii Biosciences)

*CABP* community-acquired bacterial pneumonia, *cIAI* complicated intra-abdominal infections, *cUTI* complicated urinary tract infections, *IV* intravenous, *NP* natural product, *PBP* penicillin binding protein, *po* *per orem* (oral), *NTM* non-tuberculosis mycobacteria, *S* synthetic, *uUTI* uncomplicated urinary tract infections, *UTI* urinary tract infections

^a^Compounds with new pharmacophores and MoA are underlined

**Table 4:** Compounds in, or that have recently completed, phase-II clinical trials (structures in Figs. 5–7)

Name (synonym)^a^	Compound class (lead source)	Mode of action	Administration; indication (Developer)
sanfetrinem cilexetil (**14**) (prodrug, GV-104326); sanfetrinem (**15**)	trinem (β-lactam) (NP)	PBP (cell wall)	po; TB (GSK)
MGB-BP-3 (**16**)	distamycin A (NP)	DNA minor groove binding	po topical; CDI (MGB Biopharma)
exeporfinium chloride (**17**) (XF-73)	porphyrin (NP)	membrane-perturbing activity	topical; post-surgical nasal decolonization (Destiny Pharma)
cannabidiol (**18**) (BTX 1801)	cannabidiol (NP)	membrane disruption (cell wall)	topical, *S. aureus* infections (Botanix Pharmaceuticals)
TNP-2092 (**19**) (CBR 2092)	rifamycin -quinolizinone (ABT719) hybrid^b^ (NP-S)	RNA polymerase, DNA gyrase (GyrA) and Topo IV (ParC)	IV (po topical); ABSSSI, PJI, encephalopathy (TenNor Therapeutics)
TNP-2198 (**20**)	rifamycin-nitroimidazole hybrid^b^ (NP-S)	RNA polymerase	po topical; CDI (TenNor Therapeutics)
afabicin (**21**) (prodrug, Debio-1450); afabicin desphosphono (**22**) (Debio 1452, AFN-1252)	benzofuran naphthyridine (S)	FabI inhibition (cell wall, fatty acid biosynthesis)	IV/po; ABSSSI (Debiopharm)
peceleganan (**23**) (PL-5, V681)	cationic peptide (P)	membrane disruption (cell wall)	topical, wound infections (Jiangsu ProteLight Pharmaceutical and Biotechnology)
Recce-327^c^ (R327)	acrolein polymer (S)	disruption of cellular bioenergetics via membrane potential and/or ATP synthesis	topical, burn wound infections (Recce Pharmaceuticals); IV administration in Phase-I trial
pravibismane (**24**) (MBN-101)	bismuth thiol (S)	disruption of cellular bioenergetics via membrane potential	topical; diabetic foot infections and orthopedic-implant infection (Microbion Corporation)
DNV-3837 (**25**) (prodrug, MCB-3837); DNV-3681 (**26**)	oxazolidinone-quinolone hybrid^b^ (S-S)	protein synthesis inhibition, DNA gyrase (GyrA) and topo IV (ParC)	IV; CDI (Deinove)
ibezapolstat (**27**) (ACX-362E)^*a*^	dichlorobenzyl guanine (DCBG) (S)	DNA polymerase IIIC	po topical; CDI (Acurx Pharmaceuticals)
CRS3123 (**28**) (REP3123)	“diaryldiamine” (S);	methionyl-tRNA synthetase (protein synthesis)	po topical, CDI (Crestone)
*Mycobacteria*			
delpazolid (**29**) (RMX2001, LCB01-0371)	oxazolidinone (S)	protein synthesis inhibition	po; TB and MRSA bacteremia (LegoChem Biosciences)
sutezolid (**30**) (PF-2341272, PNU-100480)	oxazolidinone (S)	protein synthesis inhibition	po; TB (European and Developing Countries Clinical Trials Partnership / TB Alliance / Sequella)
telacebec (**31**) (Q203)	imidazo[1,2-*a*]pyridine amide (S)	respiratory cytochrome bc1 complex	po; TB (Qurient Co)
fobrepodacin (**32**) (prodrug, SPR720, pVXc-486); SPR719 (**33**)	“ethyl urea benzimidazole” (S)	DNA gyrase (GyrB) and Topo IV (ParE)	po; TB (Spero Therapeutics)
BTZ-043 (**34**)	benzothiazinone (S)	DprE1 (cell wall)	po; TB (European and Developing Countries Clinical Trials Partnership)
quabodepistat (**35**) (OPC-167832)	3,4-dihydrocarbostyril (S)	DprE1 (cell wall)	po; TB (Otsuka Pharmaceutical)
GSK3036656 (**36**)	oxaborole (S)	leucyl-tRNA synthetase (protein synthesis)	po; TB (GSK)
TBA-7371 (**37**)	azaindole (S)	DprE1 (cell wall)	po; TB (TB Alliance / Foundation for Neglected Disease Research / Bill & Melinda Gates Medical Research Institute)
sudapyridine (**38**) (WX-081)	diarylquinoline (S)	mycobacterial ATP synthase inhibition	po; TB (Shanghai Jiatan Biotech)
pyrifazimine (**39**) (TBI-166)	riminophenazine (clofazimine) (S)	DNA binding leading to cell cycle disruption	po; TB (Institute of Materia Medica / Chinese Academy of Medical Sciences / Peking Union Medical College)
*Non-traditional small molecules*			
fluorothiazinon (**40**) + cefepime (**41**)	thyazinone (S) + cephalosporin (NP)	bacterial type III secretion system (T3SS)	po; G-ve virulence (Gamaleya Research Institute of Epidemiology and Microbiology)
dovramilast (**42**) (CC-11050, AMR-634)	“3-oxo-1H-isoindol-4-yl” (S)	phosphodiesterase 4 (PDE4) inhibitor (host immune response)	po, Leprosy and TB (Medicines Development for Global Health)

*ABSSSI* acute bacterial skin and skin structure infections, *CDI* *C. difficile* infection, *IV* intravenous, *NP* natural product, *po* *per orem* (oral), *PBP* penicillin binding protein, *PJI* prosthetic joint infections, *P* protein/mammalian peptide, *S* synthetic, *TB* tuberculosis

^a^Compounds with new pharmacophores and MoA are underlined

^b^Individual components of these hybrid antibacterials have known MoAs

^c^Recce-327 is a polymer and its structural units have not been disclosed

**Table 5:** Compounds in phase-I clinical trials (structures in Figs. 8–10)

Name (synonym)^a^	Compound class (lead source)	Mode of action^a^	Administration; indication (developer)
SPR206 (**43**)	polymyxin (NP)	membrane disruption (cell wall)	IV; G-ve (Spero Therapeutics)
MRX-8^b^	polymyxin (NP)	membrane disruption (cell wall)	IV; G-ve (MicuRx Pharmaceuticals)
QPX-9003 (**44**)	polymyxin (NP)	membrane disruption (cell wall)	IV; G-ve (Qpex Biopharma)
RG6319^b^	“arylomycin” (NP)	type I signal peptidase (LepB) inhibitor (cell wall)	not disclosed; G-ve, cUTI (Genentech)
zifanocycline (**45**) (KBP-7072)	tetracycline (NP)	protein synthesis inhibition	IV/po; G-ve and G+ve (KBP Biosciences)
apramycin (**46**) (EBL-1003)	aminoglycoside (NP)	protein synthesis inhibition	IV; G-ve (Juvabis)
PLG0206 (**47**) (WLBU2)	cationic peptide (P)	membrane disruption (cell wall)	topical; G-ve and G+ve, PJI (Peptilogics) / IV administration in phase-I trial
PL-18 (**48**)	cationic peptide (P)	membrane disruption (cell wall)	topical (suppository), G-ve and G+ve, bacterial vaginosis (Jiangsu ProteLight Pharmaceutical and Biotechnology)
murepavadin (**49**) (POL7080)	protegrin I (P)	β-barrel protein LptD (Imp/OstA) inhibition (cell wall)	inhalation; pseudomonal infections (G-ve) (Spexis)
TXA709 (**50**) (prodrug); TXA707 (**51**)	FtsZ benzamide (S)	FtsZ inhibition (cell wall)	po; G+ve (TAXIS Pharmaceuticals)
RG6006^b^ (RO7223280)	macrocyclic peptide (S)	unknown	IV, *A. baumannii* infections (Roche)
BWC0977 (**52**)	oxazolidinone containing NBTI (S)	DNA gyrase and topoisomerase IV	IV/po, G-ve and G+ve, but being developed for G-ve (Bugworks Research)
*Mycobacteria*			
macozinone (**53**) (PBTZ 169)	benzothiazinone (S)	DprE1 (cell wall)	po; TB (Innovative Medicines for Tuberculosis Foundation / Nearmedic Plus)
TBI-223 (**54**)	oxazolidinone (S)	protein synthesis inhibition	po; TB (TB Alliance / Institute of Materia Medica)
TBAJ-876 (**55**)	diarylquinoline (S)	mycobacterial ATP synthase inhibition	po; TB (TB Alliance)
TBAJ-587 (**56**)	diarylquinoline (S)	mycobacterial ATP synthase inhibition	po; TB (TB Alliance)
GSK2556286 (**57**) (GSK-286)	“uracil aryloxypiperidine” (S)	complex pathway related to cholesterol catabolism (adenylyl cyclase Rv1625c)	po, TB (GSK)
*Non-traditional small molecules*			
BVL-GSK098 (**58**) + ethionamide (**59**)	spiroisoxazoline (S)	inactivation of TetR-like repressor (EthR2) – ‘resistance breaker’	po, TB (BioVersys / GSK)
GSK3882347^b^	mannose-derived (S)	Type 1 fimbrin D-mannose specific adhesin (FimH) antagonist – antivirulence	po, G-ve, UTI (GSK / Fimbrion Therapeutics)
ALS4^b^	undisclosed (S)	4,4ʹ-diapophytoene desaturase (CrtN, staphyloxanthin biosynthesis) – antivirulence	po, G+ve, *S. aureus* infections (Aptorum Therapeutics)

*G-ve* Gram-negative, *G+ve* Gram-positive, *IV* intravenous, *NP* natural product, *PJI* prosthetic joint infections, *NBTI* novel bacterial topoisomerase inhibitor, *P* protein/mammalian peptide, *PBP* penicillin binding protein, *po* *per orem* (oral), *S* synthetic, *TB* tuberculosis

^a^Compounds with new pharmacophores and MoA are underlined

^b^Structures not publicly disclosed

**Table 6:** β-Lactam/β-lactamase inhibitor (BL/BLI) combinations in clinical trials or submitted for regulatory approval (structures in Figs. 11–13)

Name (synonym)^a^	Compound (lead source)	Administration; indication (developer)
*NDA/MAA*		
durlobactam (ETX2514) (**60**) + sulbactam (**61**)	DBO BLI (S)^b^ + clavulanic acid (NP)	IV; MDR *Acinetobacter* infections (Innoviva)
*Phase-III*		
taniborbactam (**62**) (VNRX-5133) + cefepime (**41**)	bicyclic boronate BLI (S) + cephalosporin (NP)	IV; cUTI (VenatoRx Pharmaceuticals)
enmetazobactam (**63**) (AAI 101) + cefepime (**41**)	clavulanic acid (NP) + cephalosporin (NP)	IV; UTI (Allecra Therapeutics)
zidebactam (**64**) + cefepime (**41**)	DBO BLI (S)^b^ & PBP2 + cephalosporin (NP)	IV; G-ve (Wockhardt)
*Phase-I*		
nacubactam (**65**) (OP0595) + meropenem (**66**)	DBO BLI (S)^b^ + carbapenem (NP)	IV; G-ve (NacuGen Therapeutics)
xeruborbactam (**67**) (QPX7728) + QPX2014^c^QPX7831 (**68**) (prodrug) + QPX2015^c^	bicyclic boronate BLI (S) + BL (NP)	IV/po^d^; G-ve (Qpex Biopharma)
ETX0282 (**69**) (prodrug) + cefpodoxime proxetil (**70**) (prodrug); ETX1317 (**71**) + cefpodoxime (**72**)	DBO BLI (S)^b^ + cephalosporin (NP)	po; G-ve, UTI (Innoviva/CARB-X)
ledaborbactam etzadroxil (**73**) (prodrug, VNRX-7145) + ceftibuten (**74**); ledaborbactam (**75**)	bicyclic boronate BLI (S) + cephalosporin (NP)	po; G-ve (VenatoRx Pharmaceuticals)
funobactam (XNW-4107) (**76**) + imipenem (**77**) + cilastatin (**78**)	DBO BLI (S) + carbapenem (NP)	IV; G-ve (Sinovent/Evopoint Biosciences)
PF-07612577 (CTB + AVP) [AVP (**79**) (prodrug, PF-07338233, ARX-006, ARX-1796) + ceftibuten (**74**) (PF-06264006)]	avibactam (**80**) DBO prodrug (S) + cephalosporin (NP)	po; G-ve (Pfizer)

*BL* β-lactam, *BLI* β-lactamase inhibitor, *cUTI* complicated urinary tract infections, *G-ve* Gram-negative, *IV* intravenous, *NP* natural product, *MDR* multi-drug resistant, *po* *per orem* (oral), *S* synthetic, *UTI* urinary tract infections

^a^Compounds with new pharmacophores are underlined

^b^These DBO BLIs also have activity against selected Enterobacteriaceae

^c^Structures not publicly disclosed

^d^Xeruborbactam (**67**) is administered IV and its prodrug QPX7831 (**68**) is administered po, but this is counted once as IV/po in Tables S1 and S2

**Table 7:** Compounds discontinued or likely to have been discontinued from clinical development since 2018 or the previous review [23]

Name (synonym) [References]	Compound class (lead source); mode of action	Last known status and indication
iclaprim [374]	trimethoprim (S); dihydrofolate reductase (DHFR)	NDA, IV/po; ABSSSI (Motif Bio) – FDA required an additional phase-III trial and development was halted
SQ109 [375–378]	“ethambutol analog” (S); mycolic acid transporter inhibitor (cell wall)	po; TB (Sequella) – a Russian phase II/III trial was completed in 2017 by Infectex [362, 363]; funding for further development is being sought by Sequella (Rockville, MD, USA)
ridinilazole (SMT 19969) [379–382]	bis-benzimidazole (S) [potential new class]; cell division inhibitor	Phase-III; po topical; CDI (Summit) – ridinilazole was non-superior compared to vancomycin in terms of clinical response, and an additional phase-III trial would have been required [374]
ancremonam (BOS-228, LYS228) [383, 384]	monobactam (NP); PBP (cell wall)	IV; cUTI and cIAI (Boston Pharmaceuticals) – licensed by Novartis to Boston Pharmaceuticals (Cambridge, MA, USA) in July 2018, but there has been no further development
OPS-2071 [385, 386]	fluoroquinolone (S); DNA gyrase	po topical; CDI (Otsuka) – discontinued in 2020 for CDI and Crohn’s Disease
TP-6076 [387, 388]	tetracycline (NP); protein synthesis inhibition	IV; G-ve (Tetraphase) – phase-I trial (NCT03691584) was completed in December 2019 and there has been no further development; Tetraphase was acquired by La Jolla Pharmaceutical in July 2020, who are now part of Innoviva (Burlingame, CA, USA)
TP-271 [389–391]	tetracycline (NP); protein synthesis inhibition	IV/po; G+ve/ G-ve (Tetraphase) – phase-I trials (e.g., NCT03024034) completed in 2018 and there has been no further development; Tetraphase was acquired by La Jolla Pharmaceutical in July 2020, who are now part of Innoviva (Burlingame, CA, USA)
SPR741 + β-lactam [392, 393]	polymyxin (NP); membrane permeabilizer (cell wall)	IV; G-ve (Spero) – phase-I trials (NCT03022175 and NCT03376529) completed in 2017 and development was discontinued in 2020
GT-1 (LCB10 0200) [394, 395]	cephalosporin siderophore (NP); PBP (cell wall)	IV (Geom) – phase I trial (ACTRN12618001980224) started in March 2019 but was halted due to safety concerns
BCM-0184	not disclosed	po (& topical); G+ve (Biocidium) – phase-I trial was not registered and there has been no update
niclosamide (ATx201) [396]	salicylanilide (S); oxidative phosphorylation; quorum sensing	topical; *H. pylori*; impetigo (Union Therapeutics) – phase-II trials (NCT03009734 and NCT03429595) finished in 2018; only listed in their pipeline for COVID-19 prophylaxis (NCT04932915)
auranofin [236]	auranofin (S); thiol-redox homeostasis	po; TB (The Aurum Institute) – phase-II trial (NCT02968927) has not been updated since January 2019
DSTA4637S [397, 398]	mAb rifamycin conjugate (ADC); RNA synthesis	IV; G+ve (Genentech) – phase-I trial (NCT03162250) completed in January 2020 but DSTA4637S is not listed on their pipeline

*ABSSSI* acute bacterial skin and skin structure infections, *ADC* antibody drug conjugate, *CDI* *C. difficile* infections, *cIAI* complicated intra-abdominal infections, *cUTI* complicated urinary tract infections, *G-ve* Gram-negative, *G+ve* Gram-positive, *NP* natural product, *PBP* penicillin binding protein, *S* synthetic, *TB* tuberculosis

**Table 8:** New antibacterial pharmacophores, separated into major MoA classes, including compound name, phase, antibacterial class, lead source, activity, MoA and administration

Name – phase	Class (lead source)	Mode of action^a^ (target) - administration
taniborbactam (**62**) – III	bicyclic bornonate (S)	cell wall (BLIs) – IV
xeruborbactam (**67**) – I	bicyclic bornonate (S)	cell wall (BLIs) – IV and po (prodrug)
ledaborbactam etzadroxil (**73**) – I	bicyclic bornonate (S)	cell wall (BLIs) – po (prodrug)
afabicin (**21**) – II	benzofuran naphthyridine (S)	cell wall, fatty acid biosynthesis (FabI) – IV and po (prodrug)
quabodepistat (**35**) – II	3,4-dihydrocarbostyril (S)	cell wall (DprE1) – po
BTZ-043 (**34**) – II	benzothiazinone (BTZ) (S)	cell wall (DprE1) – po
macozinone (**53**) – I	benzothiazinone (BTZ) (S)	cell wall (DprE1) – po
TBA-7371 (**37**) – I	azaindole (S)	cell wall (DprE1) – po
TXA709 (**50**) – I	FtsZ benzamide (S)	cell wall (FtsZ) – po (prodrug)
murepavadin (**49**) – I	“protegrin” (P)	cell wall (LptD) – IV & inhalation
RG6319 – I	“arylomycin”^b^ (NP)	cell wall, protein transport (SPase 1) – not disclosed
exeporfinium (**17**) – II	porphyrin (NP)	cell wall/membrane perturbation – topical
cannabidiol (**18**) – II	cannabidiol (NP)	cell wall/membrane perturbation – topical
peceleganan (**23**) - II	cationic peptide (P)	cell wall/membrane perturbation – topical
PLG0206 (**47**) – I	cationic peptide (P)	cell wall/membrane perturbation – topical/IV
PL-18 (**48**) – I	cationic peptide (P)	cell wall/membrane perturbation – topical
Recce-347 – I	polymer (S)	cell wall/membrane perturbation – topical/IV
gepotidacin (**10**) – III	triazaacenaphthylene (S)	DNA (GyrA) – IV/po
zoliflodacin (**11**) – III	spiropyrimidinetrione (S)	DNA (GyrB) – po
MGB-BP-3 (**16**) – II	distamycin (NP)	DNA (minor groove binding) – po topical
ibezapolstat (**27**) – II	dichlorobenzyl guanine (S)	DNA (DNA polymerase IIIC) – po topical
fobrepodacin (**32**) – II	“ethyl urea benzimidazole” (S)	DNA (GyrB and ParE) – po (prodrug)
BWC0977 (**52**) – I	“oxazolidinone containing NBTI” (S)	DNA (DNA gyrase and topoisomerase IV) - I and po
epetraborole (**13**) – II/III	oxaborole (S)	protein synthesis (leucyl-tRNA synthetase) – po
GSK3036656 (**36**) – II	oxaborole (S)	protein synthesis (leucyl-tRNA synthetase) – po
CRS3123 (**28**) – II	“diaryldiamine” (S)	protein synthesis (methionyl-tRNA synthetase) – po topical
*Antibacterials with other MoA*		
telacebec (**31**) – II	imidazo[1,2-*a*]pyridine amide (S)	oxidative phosphorylation (respiratory complex bc1) – po
GSK2556286 (**57**) – I	“uracil aryloxypiperidine” (S)	cholesterol catabolism (adenylyl cyclase Rv1625c) – po
RG6006 – I	macrocyclic peptide (S)	not disclosed - IV
dovramilast (**42**) – II	“3-oxo-1H-isoindol-4-yl” (S)	anti-inflammatory (PDE4 inhibitor) – po
fluorothyazinone (**40**) – II	thyazinone (S)	antivirulence (type III secretion system) – po
GSK3882347 – I	mannose-derived (S)	antivirulence (FimH antagonist) – po
ALS4 – I	not disclosed (S)	antivirulence (4,4ʹ-diapophytoene desaturase, CrtN) – po
BVL-GSK098 (**58**) – I	spiroisoxazoline (S)	resistance reversal (inactivation of TetR-like repressor) - po

^a^New MoA are Underlined

^b^Likely structure class

Data in this review were obtained by analyzing the scientific literature and internet sources such as company and funding organization websites, clinical trial registers, The Pew Charitable Trusts (Philadelphia, PA, USA) [28] and World Health Organization (WHO) (Geneva, Switzerland) pipeline analyses [21, 22] and biotechnology newsletters. Every effort has been made to ensure the accuracy of this data; however, it is possible that compounds in the early stages of clinical development have been overlooked as there is limited information available in the public domain.

## Antibacterial drugs launched from January 2013 to December 2022

In the last 10 years, 19 new small molecule antibacterial drugs (eight NP-derived and 11 synthetic-derived) and four new BL/BLI combinations have been approved (Table 1 and S1, Figs. 1 and 2). Among these 19 antibacterial drugs, none was first-in-class, with the last being bedaquiline in 2012 (diarylquinoline class), which also was the first new tuberculosis (TB) drug class since 1963 [38]. Although the semi-synthetic pleuromutilin derivative lefamulin was approved in 2019 for systemic use for community-acquired bacterial pneumonia (CABP) infection, a topically administered pleuromutilin, retapamulin, was approved in 2007. While new classes of G-ve antibacterial drugs have been approved, new exemplars within existing classes, especially BL/BLI combinations, also show improved activity profiles against resistant G-ve bacteria.

Since the 2019 review [24] in this series, two new small molecule antibacterials (Table 1, Figs. 1 and 2), levonadifloxacin (**1**) (as its prodrug alalevonadifloxacin (**2**)) and contezolid acefosamil (**3**) were first approved in India and China respectively.

Levonadifloxacin (**1**) (Emrok, WCK-771; IV), which is the arginine salt of the fluoroquinolone *S*-(–)-nadifloxacin, and its alanine prodrug alalevonadifloxacin (**2**) (Emrok O, WCK-2349; po) [39–41] were developed by Wockhardt (Mumbai, Republic of India). Both the IV and oral formulations were approved in January 2020 by the Indian Central Drugs Standard Control Organization (CDSCO) for the treatment of acute bacterial skin and skin structure infections (ABSSSI), including diabetic foot infections and concurrent bacteremia [42, 43]. Levonadifloxacin (**1**) has activity against G+ve bacteria including MRSA, as well as some G-ve bacteria [41], and a prescription-event monitoring study was recently published [44]. Racemic nadifloxacin was first approved in 1993 to topically treat acne and MRSA infections [45].

Contezolid acefosamil (**3**) (Youxitai, MRX-4; po) is an oxazolidinone prodrug developed by MicuRx Pharmaceuticals (Hayward, CA, USA and Shanghai, People’s Republic of China). It was approved by the Chinese National Medical Products Administration (NMPA) in June 2021 for the treatment of complicated skin and soft tissue infections (cSSTI), including, but not limited to, MSSA, MRSA, *Streptococcus pyogenes* and *Streptococcus agalactiae* [46–48]. The development pathway from contezolid (**4**) (MRX-I) [49] to **3** was recently published [50]. The prodrug provides dramatic improvements in solubility over the parent antibiotic (from 0.2 mg ml^−1^ to >200 mg ml^−1^), leading to exposure of contezolid (**4**) in rats after IV administration of contezolid acefosamil (**3**) like, or higher than, that from direct IV administration of **4**. A phase-III trial (NCT05369052) evaluating contezolid acefosamil (**3**) (po)/contezolid (**4**) (IV) for diabetic foot infections compared to linezolid began in May 2022.

Three non-traditional antibacterial drugs (two mAbs and one biotherapeutic) were launched between 2013 and 2022 to treat bacterial infections (Table 2), compared to 19 traditional antibacterial drugs launched during this period (Table 1).

Obiltoxaximab [51, 52] is a mAb that neutralizes harmful toxins produced by *Bacillus anthracis* that was approved using the US FDA Animal Rule based on their efficacy in relevant animal models and safety in phase-I studies. Another mAb that also neutralizes *B*. *anthracis* toxins, raxibacumab [52, 53], was similarly approved in 2012. The mAb bezlotoxumab, which binds to toxin B produced by *C. difficile* [54, 55], was approved in 2016 to help prevent the recurrence of CDI after successfully completing two phase-III trials [56, 57].

In November 2022, a live biotherapeutic product, RBX2660 (Rebyota), was approved by the US FDA [58] to help prevent CDI following antibiotic treatment, based on phase-III trial data [59]. RBX2660 is a liquid suspension donor fecal microbiota that has been screened for bacterial, viral and parasitic pathogens [60, 61] that was developed by Rebiotix Inc (Roseville, MN, USA), which is part of Ferring Pharmaceuticals (Saint-Prex, Switzerland). There is also another phase-III trial (NCT03931941) in progress.

Although outside the cut-off period, another non-traditional antibacterial product, Vowst (SER-109), developed by Seres Therapeutics Inc (Cambridge, MA, USA) and Nestlé Health Science (Hoboken, NJ, USA) was approved by the US FDA on 26 April 2023.^1^

## Compounds undergoing clinical evaluation

Direct acting small molecules, mammalian-derived peptides and polymeric compounds currently undergoing clinical trials or under regulatory evaluation for the treatment of bacterial infections on 31 December 2022 are detailed in the following tables and figures: NDA and phase-III in Table 3 and 6 with structures in Figs. 3, 4, 11, and 12, phase-II in Table 4 with structures in Figs. 5–7, and phase-I in Tables 5 and 6 with structures in Figs. 8–10 and 13. Non-traditional antibacterial candidates that are not small molecules such as biotherapeutic microbiome modulation, phage therapy, and antibodies have not been included in this review.

### Compounds in NDA/MAA filing (Table 3, Fig. 3)

Solithromycin (**5**) (T-4288, CEM-101; IV/po) is a semi-synthetic 2-fluoroketolide [62] that is being developed by FUJIFILM Toyama Chemical Co., Ltd. (Tokyo, Japan). In April 2019, an NDA was submitted to the Japanese Pharmaceuticals and Medical Devices Agency (PDMA) for use of **5** as a treatment for otorhinolaryngological bacterial infections. Although there have been no subsequent updates, **5** is still listed on their November 2022 pipeline as ‘NDA filing’ for otorhinolaryngology and as phase-III for respiratory infectious disease [63]. Solithromycin (**5**) was previously being developed in the USA and Europe for CABP but development was halted in 2016 and 2017 respectively [64].

### Compounds in phase-III trials (Table 3, Fig. 4)

Sulopenem (**6**) (CP-70,429), which is a synthetic thiopenem BL first developed by Pfizer (New York, NY, USA) in the 1990s [65–68], and its prodrug sulopenem etzadroxil (**7**) (PF-03709270; po) are being developed as treatments for G-ve infections by Iterum Therapeutics (Dublin, Ireland). To date, three phase-III trials have been completed and have reported results: complicated intra-abdominal infections (cIAI) (NCT03358576), cUTI (NCT03357614) [69] and uUTI (NCT03354598) [70]. In November 2020, Interim filed an NDA for uUTIs with the FDA [71] for orally administered sulopenem etzadroxil (**7**) in combination with probenecid (**8**) [72]. Probenecid (**8**) is a marketed drug for gout and hyperuricemia that increases uric acid production, which inhibits BL tubular renal secretion that leads to a longer antibiotic half-life and higher serum concentrations [73]. However, the FDA issued a Complete Response Letter (CRL) in July 2021 that indicated that the NDA was not approvable in its present form [74]. In response to this CRL, Iterum initiated another phase-III trial (NCT05584657) in October 2022 to investigate sulopenem etzadroxil (**7**) + probenecid (**8**) compared to amoxicillin + clavulanic acid for uUTI, which is scheduled to finish in March 2024.

Nafithromycin (**9**) (WCK 4873; po) is an orally bioavailable ketolide being developed by Wockhardt Limited (Mumbai, Republic of India) that is being evaluated in a phase-III trial (CTRI/2019/11/021964) in India as an oral treatment for CABP. Nafithromycin (**9**) has broad spectrum antibacterial activity against G+ves such as *S. pneumoniae* and *S. aureus* and G-ves such as *Haemophilus influenzae*, *Moraxella catarrhalis*, *Legionella pneumophila*, *Mycoplasma pneumoniae* and *Chlamydophila pneumoniae* [75–78].

Gepotidacin (**10**) (GSK-2140944; po) is a new chemotype bacterial Type II topoisomerase inhibitor [79] (new triazaacenaphthylene class) being developed by Glaxo-SmithKline (GSK) (London, UK) for uUTI and gonorrhea. In November 2022, GSK announced that two phase-III trials (NCT04020341 and NCT04010539) for cUTI were stopped early for efficacy (positive news!), with an NDA planned for the first half of 2023 [80]. Gepotidacin (**10**) is also being evaluated in another cUTI phase-III trial with Japanese participants (NCT05630833), as well as a phase-III trial against uncomplicated urogenital gonorrhea caused by *Neisseria gonorrhoeae* (NCT04010539). Gepotidacin (**10**) has activity against a range of both G+ve and G-ve pathogens [81–83], including *Mycobacteria* [84], *Stenotrophomonas maltophilia* [85], *Mycoplasma* and *Ureaplasma* [86].

Zoliflodacin (**11**) (ETX0914, AZD0914; po) is another new chemotype topoisomerase inhibitor [87] (new spiropyrimidinetrione class) being developed by Entasis Therapeutics (Waltham, MA, USA), which was recently acquired by Innoviva (Burlingame, CA, USA) [88]. Zoliflodacin (**11**) is being evaluated in a phase-III trial (NCT03959527) as an oral treatment for uncomplicated gonorrhea [89–91] in partnership with the Global Antibiotics Research and Development Partnership (GARDP) (Geneva, Switzerland). GARDP has the right to register and commercialize **11** in low- and middle-income countries [92]. Zoliflodacin (**11**) also has activity against *Mycoplasma genitalium*, which could broaden its effectiveness as a treatment for sexually transmitted infections [93].

Benapenem (**12**) (IV) is a carbapenem that completed a phase-II/III trial in May 2020 (NCT04505683) as an intravenous treatment for cUTI, including pyelonephritis, by Sihuan Pharmaceutical (Beijing, People’s Republic of China). Benapenem (**12**) is structurally related to ertapenem and has a similar extended human half-life of 7 h, which supports once-daily IV dosing like ertapenem, an advantage over other carbapenems that require multiple daily dosing due to shorter half-lives [94, 95].

Epetraborole (**13**) (GSK2251052, AN3365, and BRII-658; po) is a benzoxaborole leucyl-tRNA synthetase (LeuRS) inhibitor [96], which is a new antibacterial target, being evaluated by AN2 Therapeutics (Menlo Park, CA, USA) in a phase-II/III (NCT05327803) against treatment-refractory *Mycobacterium avium* complex (MAC) lung disease. MAC accounts for up to 85% of non-tuberculosis mycobacteria (NTM) related lung disease [97]. Epetraborole (**13**) has also been reported to have in vivo activity against *Mycobacterium abscessus*, another NTM involved in lung infections [98, 99]. Epetraborole (**13**) was originally developed as a treatment for G-ve infections in phase-II trials for cUTI (NCT01381549) and cIAI (NCT01381562) but these studies were halted due to resistance developing in patients during the cUTI trial [100]. Brii Biosciences (Durham, NC, USA and Shanghai, People’s Republic of China) have licensed **13** for development in the Greater China region [101].

### Traditional antibacterial compounds in phase-II trials (Table 4, Figs. 5 and 6)

Sanfetrinem cilexetil (**14**) (GV-104326; po) is a 1-(cyclohexyloxycarbonyloxy)ethyl ester prodrug of the trinem (tricyclic carbapenem) sanfetrinem (**15**) first developed in the 1990s by Glaxo Wellcome, which is now part of GSK (London, UK). Sanfetrinem (**15**) is active against a range of G+ve (e.g., *S. aureus*, *S. pneumoniae* and *H. influenzae*) and G-ve bacteria (e.g., *E. coli*, *M. catarrhalis*) [102–104]. Although sanfetrinem cilexetil (**14**) successfully completed a phase-II trial for respiratory infections in 1999, no further development work was undertaken until GSK started a phase-II trial (NCT05388448) in May 2022, which is evaluating **14** against rifampicin-susceptible pulmonary TB [105]. There has been a recent surge in interest in investigating carbapenem-type antibacterials as TB treatments, as evidenced by TASK (Cape Town, South Africa) leading a study that showed meropenem (**66**) in combination with amoxicillin + clavulanic acid had efficacy in a phase-II TB trial (NCT02349841) [106], as well as a consortium of private and public organizations that screened approximately 8,900 carbapenems against *Mycobacterium tuberculosis* (Mtb) [107].

MGB-BP-3 (**16**) (po topical) is a DNA binding antibacterial being developed by MGB Biopharma (Glasgow, UK) that successfully completed a phase-II trial (NCT03824795) in May 2020 for the treatment of *C. difficile*-associated diarrhea (CDAD) [108]. MGB-BP-3 (**16**) was discovered at the University of Strathclyde (Glasgow, UK) and was inspired by the actinomycetes-derived minor groove binders, distamycin, netropsin and thiazotropsin [109, 110]. In addition to activity against *C. difficile*, **16** has activity against a range of G+ve bacteria including *S. aureus* and *Enterococcus faecalis* but is not active against G-ve bacteria due to a lack of intracellular accumulation [111]. It was shown that two molecules of **16** bound to the minor groove of dsDNA, which then interfered with transcription, the supercoiling action of gyrase, and the relaxation and decatenation by topoisomerase IV enzymes in vitro [111]. This is mechanistically distinct from fluoroquinolones that cause an increase in double strand breaks, as well as induce *recA* and *lexA* SOS responses. A preprint has reported that **16** also binds to and inhibits multiple essential promoters on the *S. aureus* chromosome [112]. Furthermore, **16** is equally effective against ciprofloxacin-resistant and ciprofloxacin-susceptible strains [113].

Exeporfinium chloride (**17**) (XF-73; topical) is a photosensitizing porphyrin derivative with broad-spectrum G+ve activity [114–116] and a low propensity for developing resistance [117] being developed by Destiny Pharma (Brighton, UK). Exeporfinium chloride (**17**) successfully completed a phase-II trial (NCT03915470) in March 2021 that investigated its activity against nasal *S. aureus* in patients at risk of post-operative infections. Destiny Pharma plans to start two phase-III nasal decolonization trials in 2024 after securing a partnering deal [118].

Synthetic cannabidiol (**18**) (CBD, BTX 1801; topical) has been evaluated in a phase-II trial (ACTRN12620000456954) by Botanix Pharmaceuticals (Perth, Australia) for the clearance of nasally colonized *S. aureus*, as well as in phase-II trials in acne (BTX 1503, NCT03573518) and atopic dermatitis (BTX 1204, NCT03824405). Cannabidiol (**18**) is the major non-psychoactive component of cannabis (*Cannabis sativa* and *C. indica*) and its G+ve antibacterial activity, along with that of the major psychoactive compound Δ^9^-tetrahydrocannabinol, was reported as having potential as a topical antibacterial in 1976 [119]. Anti-MRSA activity of **18** was later confirmed in 2008 [120] and 2020 [121] studies, along with other analogs. In 2021, an in-depth study showed that **18** was active against drug resistant strains of *S. aureus*, *S. pneumoniae*, *E. faecalis*, *Cutibacterium acnes* and *C. difficile*, less active against *S. pyogenes* and *S. agalactiae*, weakly active against *Mycobacterium smegmatis* and barely active against Mtb [122]. While cannabidiol (**18**) was inactive against *E. coli*, *Klebsiella pneumoniae*, *Pseudomonas aeruginosa* and *Acinetobacter baumannii*, it also displayed activity against four G-ve bacteria: *N. gonorrhoeae*, *Neisseria meningitidis*, *M. catarrhalis* and *L. pneumophila* [122]. It was also demonstrated that **18** was active against MSSA and MRSA biofilms, was active in topical in vivo models (though highly formulation-dependent) and that its MoA involved cytoplasmic membrane disruption [122]. It was recently shown that **18** could also act as an adjuvant with bacitracin, a cell wall inhibitor, via inhibition of undecaprenyl pyrophosphate dephosphorylation [123]. Genomic analysis demonstrated that less susceptible *S. aureus* strains contained mutations in the transporter *farE*/*farR* efflux pump system [123]. Additionally, screening of the Nebraska Transposon Mutant Library identified that strains with insertions involved in menaquinone biosynthesis had increased susceptibility to **18** that could be reversed by the addition of menaquinone [123]. The menaquinone biosynthesis pathway has been shown to be a promising drug target for *S. aureus* [124, 125].

TNP-2092 (**19**) (CBR 2092; IV) is being developed by TenNor Therapeutics (Suzhou, People’s Republic of China) and completed a phase-II trial (NCT03964493) for the treatment of G+ve ABSSSI infections using IV dosing in September 2020. TenNor have also evaluated capsule administration of **19** for hyperammonemia/hepatic encephalopathy in a phase-II trial with patients with liver cirrhosis [126, 127], while a phase-I trial (NCT04294862) for Prosthetic Joint Infection (PJI) employed IV administration [128]. TNP-2092 (**19**) is a rifamycin-quinolizinone (lead ABT-719) hybrid G+ve antibacterial discovered by Cumbre Pharmaceuticals [126, 129, 130] and its MoA is via inhibition of the targets of both antibacterial components: RNA polymerase (rifamycin) and DNA gyrase and topoisomerase IV (quinolone/quinolizinone) [131].

TNP-2198 (**20**) (IV) is another hybrid being developed by TenNor Therapeutics (Suzhou, People’s Republic of China); in this case, a rifamycin-metronidazole hybrid [132] for microaerophilic and anaerobic infections, which include gastrointestinal diseases associated with *Helicobacter pylori*, bacterial vaginosis and CDAD [133]. An *H. pylori* phase-II trial (CTR20220625 [134]) of capsules of **20** in combination with rabeprazole tablets (used to treat peptic ulcer disease) and amoxicillin capsules was completed in September 2022. An X-ray crystal structure was recently published that showed **20** bound to the rifamycin binding site on RNA polymerase with the nitroimidazole portion interacting directly with the DNA template-strand in the RNA polymerase active-center cleft, forming a hydrogen bond with a base of the DNA template strand [132]. This is supportive of RNA polymerase inhibition being involved in the MoA of **20**.

Afabicin (**21**) (Debio 1450, AFN 1720) [135–137] is a phosphate prodrug of afabicin desphosphono (**22**) (Debio 1452, AFN-1252; IV/po) being developed by Debiopharm Group (Lausanne, Switzerland). The lead compound was originally discovered by GSK (London, UK) and licensed to Affinium Pharmaceuticals, who were acquired by Debiopharm in February 2014. Afabicin (**21**) is being evaluated in a phase-II trial (NCT03723551) using an IV/oral switch strategy for the treatment of *S. aureus* bone or joint infections [138]. In an earlier phase-II trial (NCT02426918), **21** was shown to be clinically non-inferior to vancomycin/linezolid against staphylococcal ABSSSI [139]. Afabicin (**21**) specifically inhibits staphylococcal FabI [140–142], which is an essential enzyme in the final step of the fatty acid elongation cycle [143].

Peceleganan (**23**) (PL-5, V_681_; topical) is a 26-mer α-helical cationic hybrid peptide of cecropin A and melittin B [144, 145] being developed by Jiangsu ProteLight Pharmaceutical and Biotechnology (Jiangyin, People’s Republic of China). Peceleganan (**23**) is administered by spray and has successfully completed a phase-II trial in China (ChiCTR2000033334) for the treatment of bacterial wound infections [146]. No levels of **23** were detected in the patients’ blood. This indicated that there was minimal or no systemic exposure [146], a significant consideration since some cationic peptides have a history of causing nephrotoxicity. Peceleganan (**23**) has activity against both G+ve and G-ve bacteria [144, 145] and there are plans to start a phase-III trial in 2023.

Recce-327 (R327; topical and IV) is an acrolein polymer with a molecular weight range of 1–1.5 kDa [147] being evaluated by Recce Pharmaceuticals (Perth, Australia) in a phase-I/II (ACTRN12621000412831) for the treatment of G+ve and G-ve burn wound infections. A phase-I trial (ACTRN12621001313820) using IV administration of Reece-327 is being conducted with the goal of developing the polymer for serious bacterial infections such as sepsis in the future. It has been reported that the polymer disrupts bacterial cellular bioenergetics via membrane potential and/or ATP synthesis [148].

Pravibismane (**24**) (MBN-101, bismuth ethanedithiol, BisEDT; topical) is a broad spectrum antibacterial with anti-biofilm activity [149] that is being developed by Microbion Corporation (Bozeman, MT, USA). A phase-II trial (NCT05174806) evaluating **24** as a topical treatment for diabetic foot infections started in June 2022, while a phase-II trial (NCT02436876) using intraoperative administration in patients diagnosed with an orthopedic infection was completed in July 2018. This clinical work is supported by the Cystic Fibrosis Foundation (Bethesda, MD, USA) and CARB-X (Boston, MA, USA). It has been reported that **24** can cause bacterial membrane depolarization, which disrupts cellular bioenergetics [150]. Bismuth has intrinsic antibacterial activity and is a component of Pepto Bismol^®^ (bismuth subsalicylate) [151] and Xeroform^®^ (bismuth tribromophenate) [152], and is used in combination with antibiotics and a proton pump inhibitor to treat *H. pylori* infections [153]. There has recently been a resurgence in interest in the antibacterial activity of metal complexes [154, 155].

DNV-3837 (**25**) (MCB-3837; IV) is a phosphate prodrug of the oxazolidinone-quinolone hybrid DNV-3681 (**26**) (MCB-3681) being developed by Deinove (Montpellier, France). It is currently being evaluated in a phase-II CDI trial (NCT03988855) with IV administration [156]. Unfortunately, Deinove entered receivership proceedings in November 2022 [157]. The IV administration contrasts with most other antibacterials being developed for CDI, including non-traditionals [17, 21], that are almost exclusively delivered orally with little or no systemic distribution (po topical). DNV-3837 (**25**) also showed G+ve activity against MRSA, *Francisella tularensis* and *B. anthracis* [158–160].

Ibezapolstat (**27**) (ACX-362E; po topical) is a bis-substituted guanine derivative that is a bacterial DNA polymerase IIIC inhibitor [161–164] that is being evaluated in a phase-II CDI trial (NCT04247542) [165] by Acurx Pharmaceuticals (White Plains, NY, USA). DNA polymerase IIIC is a new target for clinical development and is an essential enzyme in bacteria with low guanine and cytosine content, such as *Bacillus*, *Clostridioides*, *Enterococcus*, *Mycoplasma*, *Lactobacillus*, *Listeria*, *Pneumococcus*, *Staphylococcus* and *Streptococcus* [163].

CRS3123 (**28**) (REP3123; po topical) is a methionyl tRNA synthetase (MetRS) inhibitor (new diaryldiamine class) being developed by Crestone (Boulder, CO, USA) that selectively acts on *S. aureus* and *C. difficile* MetRS with little effect on G-ve and mammalian orthologs [166, 167]. CRS3123 (**28**) prevents *C. difficile* sporulation, which leads to the inhibition of toxin production, and spares most normal gut flora [168]. CRS3123 (**28**) has completed two phase-I trials (NCT02106338 and NCT01551004) [169, 170] and is currently being evaluated in a CDI phase-II trial (NCT04781387) versus a vancomycin comparator. In the previous pipeline review [23], **28** was listed as having its development halted or discontinued. This a reminder that relatively long delays can occur in antibacterial drug development, which have been exacerbated by the COVID-19 pandemic due to disruptions to clinical trial enrollments and day-to-day operations of many organizations [171].

### Anti-mycobacterial compounds in phase-II trials (Table 4, Fig. 6)

Delpazolid (**29**) (RMX2001, LCB01-0371; po) is an oxazolidinone developed by LegoChem Biosciences, Inc. (Daejeon, Republic of Korea), which has activity against G+ve bacteria [172], Mtb [173, 174] and NTMs [175, 176]. Delpazolid (**29**) is currently being evaluated in a phase-II TB trial (NCT04550832) in combination with standard-dose bedaquiline, delamanid and moxifloxacin, compared to standard-dose bedaquiline, delamanid and moxifloxacin alone. In addition, a combination of **25** and vancomycin is being evaluated against vancomycin alone for hospitalized adults with MRSA bacteremia in a phase-IIa trial (NCT05225558). An early bactericidal activity (EBA) [177] phase-II trial (NCT02836483) showed that **29** monotherapy reduced the log-CFU of Mtb in sputum by approximately 25%, and had fewer side effects than other oxazolidinones [178].

Sutezolid (**30**) (PF-2341272, PNU-100480; po) [179] is an oxazolidinone originally developed by Upjohn & Co (later was incorporated into Pfizer (New York, NY, USA)) with activity against TB [174, 180–182] and NTMs [176]. Sequella (Rockville, MD, USA) licensed **30** from Pfizer and completed a phase-II trial (NCT01225640) in December 2011 in naive patients with drug-sensitive pulmonary TB [183]. The European and Developing Countries Clinical Trials Partnership (EDCTP; The Hague, Netherlands) is leading a phase-II trial (NCT03959566) in partnership with Sequella evaluating a combination of **30** with bedaquiline, delamanid and moxifloxacin, compared against bedaquiline, delamanid and moxifloxacin alone. The TB Alliance (New York, NY, USA) and partners [184] will also evaluate sutezolid (**30**) in a phase-II (NCT05807399) and in combination with bedaquiline and pretomanid in a phase-II/III trial (NCT05686356) later in 2023.

Telacebec (**31**) (Q203; po) is an imidazo[1,2-*a*]pyridine amide [185–187] being developed by Qurient Co., Ltd. (Seongnam-si, Republic of Korea) that completed an EBA TB phase-II trial (NCT03563599) in September 2019 [188, 189]. The imidazo[1,2-*a*]pyridine amide pharmacophore was identified during phenotypic high-content assays in infected macrophages and **31** inhibits TB growth via targeting QcrB, which is a subunit of the menaquinol cytochrome c oxidoreductase (*bc*_1_ complex) [185, 190, 191]. Telacebec (**31**) also has promise as a treatment for Buruli ulcer (*Mycobacterium ulcerans*) [192, 193].

Fobrepodacin (**32**) (SPR720, pVXc-486; po) is a DNA gyrase inhibitor phosphate prodrug being investigated by Spero Therapeutics (Cambridge, MA, USA) in a phase-II trial (NCT05496374) with patients with MAC pulmonary disease. The active metabolite SPR719 (**33**) has activity against various *Mycobacteria* [194–196] and results from a phase-I trial (NCT03796910) suggested that predicted therapeutic exposures could be attained with once-daily oral administration [197]. Fobrepodacin (**32**) and SPR719 (**33**) were originally discovered by Vertex Pharmaceuticals (Boston, MA, USA) [198–200] and inhibit DNA synthesis via bacterial gyrase (GyrB) and topoisomerase IV ParE, which is a similar MoA to novobiocin [201].

BTZ-043 (**34**) (po) is the first member of a new benzothiazinone (BTZ) class of TB antibacterials that completed a phase-I/II trial (NCT04044001) in May 2022. This study evaluated the safety, tolerability and EBA of **34**, and was led by the EDCTP (The Hague, Netherlands). BTZ-043 (**34**) inhibits the essential mycobacterial cell wall biosynthesis enzyme decaprenylphosphoryl‐β‐D‐ribose (DPR) 2′‐oxidase (DprE1) via in vivo reduction of the nitro group, generating a reactive nitroso intermediate that forms a covalent semi-mercaptal adduct with cysteine-387 [202–206]. It has been shown that BTZs can be de-aromatized in vivo through the formation of a Meisenheimer complex, which could also reduce their in vivo half-lives [207, 208]. A BTZ analog, macozinone (**53**, Fig. 9) is being evaluated in a phase-I trial.

Quabodepistat (**35**) (OPC-167832; po) is an antitubercular 3,4-dihydrocarbostyril derivative [209] being developed by Otsuka Pharmaceutical (Tokyo, Japan) that started a phase-II trial (NCT05221502) in April 2022 in combination with delamanid and bedaquiline, compared to a combination of rifampin, isoniazid, ethambutol, and pyrazinamide. Quabodepistat (**35**), which completed a phase-I/II trial in February 2022 (NCT03678688), exerts its anti-mycobacterial activity through inhibition of the cell wall synthesis enzyme DprE1 [210], which is the same target as BTZ-043 (**34**), macozinone (**53**) and TBA-7371 (**38**).

GSK3036656 (**36**) (GSK656; po) is a boron containing leucyl t-RNA synthetase inhibitor (new MoA) [211, 212] that GSK (London, UK) are currently investigating in a phase-II trial (NCT05382312) in combination with either delamanid, bedaquiline, both delamanid and bedaquiline or standard of care for 14 days in participants with newly diagnosed sputum smear positive drug-sensitive pulmonary TB. A phase-II EBA TB trial (NCT03557281) for **36** was completed in December 2021. A dechloro analog, epetraborole (**13**, Fig. 4), is currently in a phase-II/III trial (NCT05327803) against treatment-refractory MAC lung disease.

TBA-7371 (**37**) (po) is a substituted 1,4-azaindole that is being developed as a new TB treatment by the Global Alliance for TB Drug Development (New York, NY, USA), the Foundation for Neglected Disease Research (Bangalore, Republic of India) and the Bill & Melinda Gates Medical Research Institute (Cambridge, MA, USA). TBA-7371 (**37**) is currently being evaluated in a phase-II EBA and pharmacokinetic (PK) trial (NCT04176250) in patients with rifampicin-sensitive TB. TBA-7371 (**37**) is a non-covalent DprE1 inhibitor discovered by scaffold hopping from telacebec (**31**), which has a different mechanism [213–215].

Sudapyridine (**38**) (WX-081; po) is a bedaquiline analog with a chlorophenyl-methoxypyridyl group replacing the bedaquiline bromo-2-methoxy-3-quinolyl substituent [216] being developed by Shanghai Jiatan Biotech (Shanghai, People’s Republic of China). Sudapyridine (**38**) is being evaluated in a phase-II EBA trial (NCT04608955) in patients with susceptible and drug-resistant TB. Sudapyridine (**38**) has a similar in vitro and in vivo activity profile to bedaquiline, but had no adverse effects on blood pressure, heart rate, or qualitative ECG parameters during non-clinical toxicology studies [217]. Sudapyridine (**38**) also has in vitro activity against most NTM species [218].

Pyrifazimine (**39**) (TBI-166; po) is a clofazimine analog [219] (riminophenazine class) that completed a phase-II EBA TB trial (NCT04670120) in June 2021 run by the Institute of Materia Medica (Shanghai, People’s Republic of China), Chinese Academy of Medical Sciences (Beijing, People’s Republic of China) and Peking Union Medical College (Beijing, People’s Republic of China). Although clofazimine has been used to treat leprosy (*Mycobacterium leprae* infections) since 1962 and was recently incorporated into some short-course MDR-TB regimens [220, 221], its tissue accumulation can cause skin discoloration that can take months to clear. Pyrifazimine (**39**) was designed to maintain activity against TB, have improved PK/pharmacodynamics (PD) properties, and cause less skin discoloration [222–225].

### Non-traditional antibacterial compounds in phase-II trials (Table 4, Fig. 7)

Fluorothiazinon (**40**) (ftortiazinon, fluorothyazinon, C-55; po) is an orally administered inhibitor of the bacterial type III secretion system (T3SS), which is a highly conserved G-ve anti-virulence target [226] Fluorothiazinon (**40**) was developed by the Gamaleya Research Institute of Epidemiology and Microbiology (Moscow, Russia) [227–230], and has been evaluated in a phase-II trial (NCT03638830) in combination with the cephalosporin cefepime (**41**) as a potential treatment for patients with cUTI caused by *P. aeruginosa*.

Dovramilast (**42**) (CC-11050, AMG-634; po) is an isoindole phosphodiesterase type 4 (PDE4) inhibitor being developed for TB [231, 232] and leprosy type 2 reactions by Medicines Development for Global Health (Melbourne, Australia), which licensed **42** from Amgen (Thousand Oaks, CA, USA) in December 2020 [233, 234]. Dovramilast (**42**) is being evaluated in a phase-II trial (NCT03807362) at The Leprosy Mission Nepal (Katmandu, Nepal) for patients with erythema nodosum leprosum (ENL), which is an inflammatory disorder triggered by leprosy. Another phase-II trial (NCT02968927) run by The Aurum Institute NPC (Johannesburg, South Africa) has been completed [235, 236]. PDE4 inhibitors are an adjunctive host-directed therapy designed to modulate the inflammatory response to Mtb infection by reducing, but not fully blocking, TNF-α production by the host cells. The NCT02968927 trial used **42** in combination with 2HRZE/4HR therapy, which is 2 months of isoniazid (H), rifampicin (R), pyrazinamide (Z) and ethambutol (E), followed by a continuation phase of 4 months of isoniazid and rifampicin, while the NCT03807362 trial examines the safety and efficacy of CC-11050 as a monotherapy.

### Traditional antibacterial compounds in phase-I trials (Table 5, Figs. 8 and 9)

SPR206 (**43**) (IV) is a polymyxin analog being developed by Spero Therapeutics (Cambridge, MA, USA) with activity against MDR G-ve bacteria [237] and reduced nephrotoxicity compared to polymyxin. SPR206 (**43**) has completed three phase-I trials (NCT03792308, NCT04868292, and NCT04865393), with a phase-II trial planned for Q4 2023 [238]. Everest Medicines (Shanghai, People’s Republic of China) had licensed the rights for **43** in China, South Korea and several Southeast Asian countries [239], while Pfizer (New York, NY, USA) has the remaining rights outside of the USA [238].

MRX-8 (IV) is another polymyxin analog being developed by MicuRx (Hayward, CA, USA and Shanghai, People’s Republic of China) against G-ve bacteria [240–242] that completed a phase-I trial in 2021 (NCT04649541), while another phase-I is ongoing in China [243]. Although MRX-8’s structure has not been publicly disclosed, it is a polymyxin B analog with a fatty acid tail linked via a polar ester group to form a ‘soft’ prodrug [241, 244].

QPX-9003 (**44**) (F365, BRII-693; IV) is also a polymyxin derivative being developed by Qpex Biopharma (San Diego, CA, USA). It is a potential treatment for *P. aeruginosa* and *A. baumannii* infections and completed a phase-I trial in July 2022 (NCT04808414) [245]. QPX-9003 (**44**) was reported by researchers at Monash University (Melbourne, Australia) and Qpex to have reduced nephrotoxicity, acute toxicity and in vitro lung surfactant inactivation compared to other polymyxins [246]. Brii Biosciences (Durham, NC, USA and Shanghai, People’s Republic of China) have licensed QPX-9003 (**44**) for development in the Greater China region [101].

RG6319 (administration route not disclosed) is an inhibitor of LepB, which is an *E. coli* Type I signal peptidase (SPase), listed on Roche’s (Basel, Switzerland) pipeline as being evaluated in a phase-I clinical trial for cUTI [247]. SPases are enzymes that hydrolyze *N*-terminal signal peptides from proteins that are secreted across the cytoplasmic membrane and have a critical role in the viability and virulence of bacteria [248]. Although the structure of RG6319 has not been disclosed, Genetech (San Francisco, CA, USA) and The Scripps Research Institute (La Jolla, CA, USA) have been evaluating derivatives of the arylomycins, which are *Streptomyces*-derived SPase inhibitors, such as G0775 [249, 250].

Zifanocycline (**45**) (KBP-7072; IV/po) is a tetracycline derivative (aminomethylcycline) being developed by KBP BioSciences (Princeton, NJ, USA) that has completed three phase-I trials (NCT02454361, NCT02654626, and NCT04532957) and is currently being evaluated in another phase-I trial (NCT05507463). Zifanocycline (**45**) has broad spectrum antibacterial activity [251–253] and a preprint has disclosed an X-ray structure of **45** bound to the *Thermus thermophilus* 30 S ribosomal subunit [254]. As with CRS3123 (**28**), zifanocycline (**45**) was listed as discontinued or halted in the previous review [23].

Apramycin (**46**) (EBL-1003; IV) is an aminoglycoside being developed by Juvabis AG (Zurich, Switzerland) that completed a phase-I trial (NCT04105205) in October 2020. A new phase-I trial (NCT05590728) was recently started by the National Institute of Allergy and Infectious Diseases (NIAID; Rockville, MD, USA). Apramycin (**46**) has activity against carbapenem- and aminoglycoside-resistant Enterobacteriaceae, *A*. *baumannii* and *P. aeruginosa* [255, 256]. Apramycin (**46**) has been widely used as a veterinary antibiotic to treat *E. coli* and other G-ve infections [257], with European approval to treat colibacillosis and salmonellosis in calves, bacterial enteritis in pigs, colibacillosis in lambs and *E. coli* septicemia in poultry [258]. It was discovered in the 1960s at Eli Lilly & Co (Indianapolis, IN, USA) as a NP produced by *Streptomyces tenebrarius* [259, 260].

PLG0206 (**47**) (WLBU2; topical and IV) is a 24 residue membrane disrupting cationic peptide [261, 262] being evaluated by Peptilogics (Pittsburgh, PA, USA) in a phase-I trial (NCT05137314) for its potential to treat PJI in conjunction with the DAIR (debridement, antibiotics, and implant retention) surgical procedure after total knee arthroplasty. PLG0206 (**47**) has also successfully completed a phase-I trial with IV administration [263]. PLG0206 (**47**) has broad spectrum activity against G+ve and G-ve bacteria, including biofilms [261, 264, 265].

PL-18 (**48**) (HPRP-A1; topical) is a 15-mer α-helical cationic peptide derived from the *N*-terminus of the *H. pylori* ribosomal protein L1 (RpL1) that is being developed by Jiangsu ProteLight Pharmaceutical and Biotechnology (Jiangyin, People’s Republic of China). In August 2022, **48** started a phase-I trial (NCT05340790) in Australia for bacterial vaginosis using suppository administration. PL-18 (**48**) has activity against G-ve and G+ve bacteria [144, 145, 266, 267] and fungi [266], as well as induction of HeLa cell apoptosis [268] and hemolytic activity [266, 267]. These off-target activities suggest why topical administration is required for **48**.

Murepavadin (**49**) (POL7080, RG7929; inhalation) is a synthetic 14-mer cyclic peptide derived from protegrin I being developed by Spexis (Basel, Switzerland), which was formed through a merger of EnBiotix and Polyphor in December 2021. Murepavadin (**49**) has potent and selective activity against *P. aeruginosa* via binding to the *N*-terminal of the β-barrel protein LptD (Imp/OstA), a novel MoA [269–271]. Murepavadin (**49**) is reported to be in a phase-I trial for cystic fibrosis using inhaled administration [272], and was previously investigated in two phase-III trials for the treatment of *Pseudomonas* nosocomial pneumonia (NCT03582007) and VAP infections (NCT03409679). However, these trials were halted due to adverse events — an increase in serum creatinine and acute kidney injury in the nosocomial pneumonia trial in 2019 [273].

TXA709 (**50**) (po) is an anti-MRSA prodrug of TXA707 (**51**) that has been evaluated in a phase-I trial conducted by TAXIS Pharmaceuticals (Monmouth Junction, NJ, USA) [274]. TXA707 (**50**) is an inhibitor of the new antibacterial target FtsZ, which is the bacterial homolog of tubulin that plays a critical role in bacterial cell wall division in both G+ve and G-ve bacteria [275, 276]. Prolysis Ltd (Oxford, UK) originally identified PC190723 [277–279] and replacement of its Cl substituent with a CF_3_ group in TXA707 (**51**) enhanced metabolic stability, PK properties and in vivo efficacy against *S. aureus* [280, 281].

RG6006 (RO7223280, Abx MCP; IV) is being developed by Roche (Basel, Switzerland) and a phase-I trial (NCT05614895) was started in December 2022 in critically ill participants with bacterial infections using IV administration. RG6006 will be developed as a treatment for *A. baumannii* infections [247] and is a tethered macrocyclic peptide [282, 283]; however, the structure and MoA have not been publicly disclosed.

BWC0977 (**52**) (IV/po) is an oxazolidinone containing ‘novel bacterial topoisomerase inhibitor’ (NBTI) [284] with similar activity against DNA gyrase GyrA and topoisomerase IV [284–286] being developed by Bugworks Research Inc (Bangalore, Republic of India). BWC 0977 (**52**) is being evaluated in a phase-1 trial (NCT05088421) using IV administration for treating critical care G-ve infections [287, 288] with later oral step-down administration.

### Anti-mycobacterial compounds in phase-I trials (Table 5, Fig. 9)

Macozinone (**53**) (PBTZ169; po) is a benzothiazinone (BTZ) derivative [289] that was evaluated in a phase-II EBA TB trial (NCT03334734) by Nearmedic Plus LLC (Moscow, Russia), but the trial was discontinued in February 2018 due to slow enrollment. The Innovative Medicines for Tuberculosis (iM4TB) Foundation (Lausanne, Switzerland) is leading the development of **53** in the rest of the world and completed a Phase-I trial (NCT03776500) in March 2020. Macozinone (**53**) is a second generation analog of BTZ043 (**34**, Fig. 6) with the same MoA (inhibition of the mycobacterial cell wall biosynthesis enzyme DprE1) with superior physicochemical properties [289]; however, efforts have been undertaken to improve its PK and PD properties [290].

TBI-223 (**54**) (po) is an oxazolidinone [291] being developed by the TB Alliance (New York, NY, USA) and the Institute of Materia Medica (Shanghai, People’s Republic of China) that has completed two phase-I trials (NCT03758612 and NCT04865536). TBI-223 (**54**) was recently found to be active against *S. aureus* in MRSA mouse models [292].

TBAJ-876 (**55**) (po) is a bedaquiline analog (diarylquinolines class) with activity against Mtb [293] and *M. abscessus* [294], and minimal hERG channel inhibition [295, 296] that was discovered at the University of Auckland (Auckland, New Zealand). TBAJ-876 (**55**) is now being developed by the TB Alliance (New York, NY, USA) and completed a phase-I trial (NCT04493671) in November 2022, which focused on safety, tolerability, and PK. In September 2022, another phase-I trial (NCT05526911) was initiated that also evaluates its effects on CYP3A4 and P-glycoprotein. Like bedaquiline, **55** is an inhibitor of mycobacterial F-ATP synthase [297] but does not retain bedaquiline’s protonophore activity [298]. Cryogenic electron microscopy (cryo-EM) was recently used to show the binding of **55** to the F_o_ domain in *M. smegmatis* F_1_F_o_-ATP synthase [299].

TBAJ-587 (**56**) (po) is another bedaquiline analog [295] with variations in the substituents on one pyridyl ring that lead to more potent in vitro and in vivo activity against Mtb [300]. TBAJ-587 (**56**) is currently in a phase-1 trial (NCT04890535) to evaluate its safety, tolerability, and PK.

GSK2556286 (**57**) (GSK-286; po) is a substituted uracil derivative being evaluated by GSK (London, UK) in a phase-I trial (NCT04472897) as a potential TB treatment [301]. GSK2556286 (**57**) was discovered by screening against Mtb that resides within human (THP-1) macrophage-like differentiated monocytes and had an IC_50_ of 0.07 µM [302]. In addition, **57** required cholesterol to show activity in an axenic culture and resistance mutations were mapped to Mtb adenylyl cyclase (cya) Rv1625c [302–304], which has been implicated in cholesterol utilization [305]. This is a new MoA.

### Non-traditional antibacterial compounds in phase-I trials (Table 5, Fig. 10)

BVL-GSK098 (**58**) [306] (po) is the first member of a new non-traditional, anti-TB antibacterial class (spiroisoxazoline) being developed by BioVersys (Basel, Switzerland) and GSK (London, UK). BVL-GSK098 (**58**) completed a phase-I trial (NCT04654143) in May 2022. BVL-GSK098 works through inactivation of a Mtb TetR-like repressor, EthR2, which reverses ethionamide (**59**)-acquired resistance and increased basal sensitivity to **59** [307, 308]. A phase-II EBA trial (NCT05473195) is scheduled to evaluate ethionamide (**59**) with or without BVL-GSK098 (**58**) in participants with rifampicin- and isoniazid-susceptible pulmonary TB.

GSK3882347 (po) is an *E coli* Type 1 fimbrin D-mannose specific adhesin (FimH) inhibitor being evaluated by GSK (London, UK) and Fimbrion Therapeutics (St. Louis, MO, USA) with support from CARB-X (Boston, MA, USA) [309]. GSK3882347 completed a phase-I trial (NCT04488770) in May 2021 and is currently being evaluated in a Phase-Ib trial (NCT05138822) in participants with acute uUTI. A majority of UTIs are caused by uropathogenic *E. coli* (UPEC) [310], which use their type 1 pili to adhere to the cell wall via FimH adhesin [311]. Targeting the mannose-binding lectin domain of FimH prevents UPEC from binding to the bladder wall and is a promising antivirulence approach for UTI and Crohn’s Disease [312–314]. Although the structure of GSK3882347 has not been publicly disclosed, it is likely to be a mannose-derived biphenyl derivative [315].

ALS4 (po) is an *S. aureus* anti-virulence antibacterial being developed by Aptorum Therapeutics Limited (Hong Kong, People’s Republic of China) that has completed one phase-II trial (NCT05274802). Staphyloxanthin is a golden colored carotenoid with antioxidant activity that helps to neutralize reactive oxygen species (ROS) secreted by neutrophils, which protects bacteria [316, 317]. ALS4 is an inhibitor of 4,4ʹ-diapophytoene desaturase (CrtN), which is an enzyme involved in the biosynthesis of staphyloxanthin; however, although the structure of ALS4 has not been publicly disclosed, it is likely to be related to NP16 [318, 319].

## β-Lactam/β-lactamase Inhibitor (BL/BLI) Combinations Undergoing Clinical Evaluation

The discovery of the *Streptomyces*-derived BLI clavulanic acid was a significant breakthrough that rescued the use of many BL antibiotics by inactivating enzymes responsible for their destruction. There have been four new BL/BLI combinations approved since 2014 (Table 1): Zerbaxa in 2014 (contains a new cephalosporin, ceftolozane), Avycaz in 2015 (contains a new DBO-type BLI, avibactam), Vabomere in 2017 (contains a new boronate-type BLI, vaborbactam), and Recarbrio in 2019 (contains a new DBO-type BLI, relebactam), but no new combinations were approved from 2019–2022. In this section, ten new BL/BLI combinations are currently being evaluated in clinical trials or under an NDA/MAA filing are discussed (Table 6, Figs. 11–13). It should be noted that BL/BLI combinations usually move straight from phase-I into phase-III trials.

### BL/BLI combinations in NDA/MAA filing (Table 6, Fig. 11)

Durlobactam (**60**) (ETX2514) + sulbactam (**61**) (combination: SUL-DUR, ETX2514SUL; IV) is being developed by Entasis Therapeutics (a subsidiary of Innoviva, Burlingame, CA, USA) and completed a phase-III trial (NCT03894046) for treatment of infections caused by *A. baumannii-calcoaceticus* (ABC) complex [320–322] in June 2021. In this trial, SUL-DUR demonstrated statistical non-inferiority versus colistin for the primary end point of 28-day all-cause mortality in patients with carbapenem-resistant ABC infections and a significant difference in clinical cure rates, as well as a statistically significant reduction in nephrotoxicity [323]. On 17 April 2023, the US FDA Antimicrobial Drugs Advisory Committee voted 12-0 in favor of SUL-DUR for the treatment of adults with HABP/VABP caused by susceptible ABC strains.^2^ Durlobactam (**60**) is a DBO-type BLI [324–326], while sulbactam (**61**) is a clavulanic acid-type BLI first launched in 1986 that also has direct-acting antibacterial activity against *Acinetobacter* spp., but requires co-administration of another BLI to restore its activity against MDR strains.

### BL/BLI combinations in phase-III trials (Table 6, Fig. 12)

Taniborbactam (**62**) (VNRX-5133; IV) [327] + cefepime (**41**) is being developed by VenatoRx Pharmaceuticals (Malvern, PA, USA) and completed a phase-III trial (NCT03840148) in December 2021 for cUTI, including acute pyelonephritis. VenatoRx have revealed that cefepime-taniborbactam had a superior primary efficacy endpoint to the carbapenem meropenem (**66**) in this trial with a similar safety profile [328], and plan to submit an NDA to the US FDA in 2023 [329]. The taniborbactam (**62**) + cefepime (**41**) combination has activity against *E. coli*, *K pneumoniae*, carbapenemase-producing *Enterobacterales* and *P. aeruginosa* [330–332]. Taniborbactam (**62**) is a bicyclic boronate BLI [333] (new class) that is effective against both serine- and metallo-β-lactamases, including extended-spectrum β-lactamase (ESBL), OXA, KPC, NDM and VIM enzymes, but not IMP [327, 334], while cefepime (**41**) is a fourth-generation cephalosporin first approved in 1994.

Enmetazobactam (**63**) (AAI 101; IV) is a clavulanic acid-type BLI with a structure closely related to tazobactam with a methyl substituent on the tazobactam triazole ring. It has activity against ESBLs and some class A and D carbapenemases [335–337], and is being developed by Allecra Therapeutics (Weil am Rhein, Germany and Saint Louis, France). A combination of **63** and the cephalosporin cefepime (**41**) completed a phase-III trial (NCT03687255) in February 2020 for cUTI using IV administration, and successfully met criteria for non-inferiority, as well as superiority to piperacillin-tazobactam with respect to the primary efficacy outcome of clinical cure and microbiological eradication [338]. Allecra Therapeutics is planning to submit an MAA in Europe, followed by an NDA in the USA.

Zidebactam (**64**) (WCK 5107; IV) is a DBO-type BLI being developed by Wockhardt Limited (Mumbai, Republic of India) that inhibits PBPs and several β-lactamases, while enhancing BL activity [339] against *A. baumannii*, *P. aeruginosa* and CRE [340–342]. A combination of **63** and cefepime (**41**) (combination WCK 5222, FEP-ZID) started a phase-III trial (NCT04979806) in August 2022 as an IV administered treatment for cUTI and acute pyelonephritis. A phase-I trial (NCT05645757) of **63** in combination with the carbapenem ertapenem (combination WCK 6777) should commence soon, with this combination showing potent in vitro activity against many carbapenemases and β-lactamases [343].

### BL/BLI combinations in phase-I trials (Table 6, Fig. 13)

Nacubactam (**65**) (OP0595, FPI-1459, RG6080, RO7079901; IV) is a DBO-type BLI [344–346], which was developed by Meiji Seika Pharma (Tokyo, Japan). Meiji Seika and Fedora Pharmaceuticals (Edmonton, AB, Canada) had previously partnered with Roche (Basel, Switzerland) [347, 348] and several phase-I trials have been completed (Meiji Seika: NCT02134834; Roche: NCT02975388, NCT03182504), as well as two phase-I trials in combination with meropenem (**66**) (Roche: NCT02972255, NCT03174795). Nacubactam (**65**) is still listed as OP0595 on Meiji Seika’s latest pipeline [349], while Fedora’s website indicates that the combination is available for licensing [350].

Xeruborbactam (**67**) (QPX7728; IV) is a bicyclic boronate BLI [333] (new class) being developed by Qpex Biopharma (San Diego, CA, USA) that displays broad spectrum β-lactamase inhibition, including against class B and class D enzymes [351–353], as well as some intrinsic G-ve antibacterial activity [354]. An IV administered combination of **67** and an undisclosed BL (QPX2014) has completed two phase-I trials (NCT04380207 and NCT05072444) with an aim to treat serious drug resistant *Acinetobacter*, *Pseudomonas* and *Enterobacterales* infections. An orally administered xeruborbactam prodrug, QPX7831 (**68**) [355] (po), completed a phase-I trial (NCT04578873) in August 2022 and there are plans to use **68** in combination with an undisclosed oral BL (QPX2015) to treat ESBLs and carbapenem-resistant *Enterobacterales* (CRE) infections.

A combination of the DBO-type BLI ETX0282 (**69**) (po) and the cephalosporin cefpodoxime proxetil (**70**), collectively called ETX0282CPDP, was evaluated in a phase-I trial that finished in September 2019 (NCT03491748) by Entasis Therapeutics (Waltham, MA, USA), who are now a wholly owned subsidiary of Innoviva (Burlingame, CA, USA). Both ETX0282 (**69**) and cefpodoxime proxetil (**70**) are esterase-cleavable prodrugs, of ETX1317 (**71**) and cefpodoxime (**72**) respectively, and the combination is being developed to treat multidrug resistant and CRE infections [356, 357]. ETX1317 (**71**) has an (*R*)-2-(*N*-oxy)-2-fluoroacetic acid unit in place of the *N*-oxy-sulfonic acid group present in other DBOs and displays some innate G-ve activity, in addition to BLI activity [356, 357].

A ledaborbactam etzadroxil (**73**) (VNRX-7145) + ceftibuten (**74**) combination (po) [358] is being developed by VenatoRx Pharmaceuticals (Malvern, PA, USA). This combination is currently being evaluated in two phase-I trials (NCT05527834 and NCT05488678) and has previously completed two other phase-I trials (NCT04243863 and NCT04877379). Ledaborbactam etzadroxil (**73**) is an esterase-cleavable prodrug of the bicyclic boronate-type BLI (new class [333]) of ledaborbactam (**75**) (VNRX-5236) [358], while **74** is a third-generation cephalosporin first approved in 1995. The ledaborbactam etzadroxil (**73**) + ceftibuten (**74**) combination is active against clinically-derived *Enterobacterales* that express ESBLs and serine carbapenemases [359–361].

A ternary combination therapy combining funobactam (**76**) (XNW-4107) + imipenem (**77**) + cilastatin (**78**) (IV) is being developed by Suzhou Sinovent Pharmaceuticals (Sinovent) (Suzhou, People’s Republic of China). Funobactam (**76**) is a DBO-type BLI [362], while imipenem (**77**) is a carbapenem-type BL that was approved in combination with cilastatin (**78**) in 1985, as well as in combination with the DBO relebactam and **78** in 2019 [363]. Cilastin (**78**) is a renal dehydropeptidase inhibitor that reduces the rate of **77** metabolism. Funobactam (**76**) has completed two phase-I trials (NCT04482569, NCT04802863) and two phase-I trials are ongoing (NCT04801043, NCT04787562). Two phase-III trials have been announced that will evaluate the funobactam (**76**) + imipenem (**77**) + cilastatin (**78**) combination against cUTI (NCT05204368) and HABP/VABP (NCT05204563).

CTB + AVP (PF-07612577; po) is a combination of the cephalosporin ceftibuten (**74**) (PF-06264006) and the DBO-type BLI avibactam (**80**) prodrug, AVP (**79**) (PF-07338233, ARX-006, ARX-1796), under development by Pfizer (New York, NY, USA). CTB + AVP is being evaluated in a phase-I trial (NCT05554237), which started in October 2022. Avibactam (**80**) in combination with ceftazidime (Avycaz) was first approved in 2015 by the US FDA and is used to treat cIAI and cUTI [364]. AVP (**79**) was first developed by Arixa Pharmaceuticals (Palo Alto, CA, USA) [365], who were acquired by Pfizer in October 2020 [366], and a prior phase-I trial (NCT03931876) had already been completed.

## Compounds discontinued from clinical development

Compounds and BL/BLI combinations that have been discontinued from clinical development or appear to have had their development halted since the 2019 review [25] are listed in Table 7 with notes indicating any known reasons for their failure or lack of progress.

## Analysis of compounds undergoing clinical trials

### Numbers of compounds undergoing clinical evaluation and their source derivation

There were 62 antibacterial clinical candidates under clinical investigation (Figs. 14 and 15) on 31 December 2022 — ten BL/BLI inhibitor combinations and 52 small molecules, mammalian-derived peptides, and a direct acting polymer. Five of the 62 are non-traditional antibacterials that target virulence (fluorothyazinone (**40**), GSK3882347 and ALS4), resistance (BVL-GSK098 (**58**)) and host inflammation (dovramilast (**42**)) (Tables 4 and 5, Figs. 7 and 10). Of the ten BL/BLI combinations, one is in NDA/MAA (Table 6, Fig. 11), three are in phase-III (Table 6, Fig. 12) and six are in phase-I (Table 6, Fig. 13). Of the remaining 52 compounds, one is in NDA/MAA (Table 3, Fig. 3), six are in phase-III (Table 3, Fig. 4), 25 are in phase-II (Table 4, Figs. 5–7) and 20 are in phase-I (Table 5, Figs. 8–10). The source derivation of the 62 compounds was divided into 41 that were synthetically derived (S), 17 that were NP derived (NP), and four that were protein/mammalian peptide derived (P) (Fig. 14).

While there was a similar number of compounds in the different development phases in 2011, 2013 and 2015 analyses (except for a reduced number in phase-III trials in 2011 (6) compared to 2013 (16) and 2015 (15)), the number in phase-I trials increased to 22 in 2019 [23] from an average of 12 compounds in 2011-2015 [24–26], and this was even higher at 26 in 2022 (Fig. 15). The number of compounds in phase-II also increased (from 18 in 2019 to 25 in 2022), reflecting the successful progression of several of the 2019 phase-I candidates and the entry of new antibacterials. At least 18 of the 26 phase-I compounds target G-ve bacteria (11 traditional compounds, one anti-virulence and six BLI combinations), with four of these also possessing G+ve activity, while there are an additional eight with G+ve only activity (six against TB and two against MRSA). While the overall numbers are still low compared to other therapeutic disease indications, the clinical pipeline is now starting to resemble the more traditional progression of attrition, rather than the flat or inverse progressions seen in 2011 [26], 2013 [25] and 2015 [24], and this likely reflects the success of push incentives driving innovative antibiotic discovery [6–10].

### New antibacterial pharmacophore analysis

A pharmacophore is the common subunit of active molecules that interact with biological targets. It is crucial to develop new antibacterial drugs with new MoA and/or pharmacophores to slow down drug resistance and to potentially allow the identification of new combination therapies. This is also why there is considerable excitement around the potential of non-traditional antibacterials, along with the yet-to-be-proven hypothesis that some modalities, such as antivirulence strategies, will not lead to resistance since bacterial survival is not directly targeted [17, 18, 20, 21].

In this review, new pharmacophores not previously found in human antibacterial drugs have been analyzed as a measure of antibacterial structure innovation (Table 8). In Table 8, compounds with new MoA not previously found in previously approved antibacterial drugs are underlined. The MoA of most traditional small molecule antibacterial drugs can be categorized into four major ‘macro’ level classes: cell wall, protein synthesis, DNA synthesis, and RNA synthesis inhibitors [367]. There are 34 different compounds — 15 in phase-I, 15 in phase-II and 4 in phase-III/NDA (Fig. 16) — and this total is significantly higher than identified in previous reviews: 11 in 2011 [26], 17 in 2013 [25], 15 in 2015 [24] and 19 in 2019 [23] (Fig. 17). Twenty-six of these compounds target the well-established ‘macro’ targets: cell wall (17), DNA (6) and protein synthesis inhibition (3). There are no novel RNA synthesis inhibitors in clinical development. Since the 2019 review [23], the boronate BLI class has expanded with the bicyclic boronates class, which includes taniborbactam (**60**), xeruborbactam (**65**) and ledaborbactam etzadroxil (**71**), now considered to be a new class [21, 22].

Existing antibacterial classes that inhibit the bacterial cell envelope include the BL, glycopeptide, polymyxin, daptomycin (lipopeptide), fosfomycin, and cycloserine classes. The new cell envelope acting antibacterials inhibit several different targets (LptD: murepavadin (**49**), FabI: afabicin (**21**), 3 × DprE: BTZ-043 (**34**) and macozinone (**53**), quabodepistat (**35**) and TBA-7371 (**37**), and FtsZ: TXA709 (**62**)) and six perturb bacterial membranes through less defined mechanisms (exeporfinium (**17**), cannabidiol (**18**), Recce-347, and the three cationic peptides, peceleganan (**23**), PLG0206 (**47**) and PL-18 (**48**)) (Table 8). Although the structure of RG6319 has not been disclosed, it is likely to be an arylomycin derivative that inhibits *E. coli* Type 1 signal peptidase, which is a key enzyme in transporting enzymes across the cytoplasmic membrane to the outer cell wall [248].

The (fluoro)quinolone class are DNA synthesis inhibitors (DNA gyrase GyrA and topoisomerase IV parC [201]) that are routinely used in clinical practice, while novobiocin, which is a DNA gyrase GyrB and topoisomerase IV ParE inhibitor, was briefly used as an antibacterial over 50 years ago [201, 368]. BWC0977 (**52**) belongs to a new antibacterial class and equally inhibits both DNA gyrase GyrA and topoisomerase IV. Fobrepodacin (**32**) is an ‘ethyl urea benzimidazole’ that also binds to both GyrB and ParE, gepotidacin (**10**) inhibits GyrB at a different binding site to the quinolones, and zoliflodacin (**11**) inhibits GyrB. Ibezapolstat (**27**) is the first member of the dichlorobenzyl guanine class that inhibits DNA polymerase IIIC, while MGB-BP-3 (**16**) is a DNA minor groove binder.

Bacterial protein synthesis inhibition can be caused by several compound classes including macrolides, aminoglycosides, tetracyclines, lincosamides, chloramphenicol, oxazolidinones, pleuromutilins and fusidic acid. There are two oxaborole-type leucine tRNA synthetase (LeuRS) inhibitors, epetraborole (**13**) and GSK3036656 (**52**), and one methionyl-tRNA synthetase inhibitor, CRS3123 (**28**), in clinical trials. The only marketed inhibitor of a tRNA synthetase is mupirocin, which targets isoleucyl-tRNA synthetase.

There are two direct-acting traditional and five non-traditional antibacterial compounds with new mechanisms. Telacebec (**31**) is an inhibitor of the mycobacterial respiratory cytochrome bc_1_ complex [185, 186, 369]. Inhibition of bacterial respiratory systems is an emerging MoA [369, 370] with three bedaquiline analogs, sudapyridine (**38**), TBAJ-876 (**55**) and TBAJ-587 (**56**) that are also in clinical development. GSK2556286 (**57**) was recently disclosed to be an adenylyl cyclase Rv1625c agonist, which interferes with cholesterol catabolism and reduces the levels of this critical carbon source [304]. RG6006 is a new antibacterial class but there is only limited public information available about the structure and MoA. There are three antivirulence compounds that employ totally different anti-virulence mechanisms: fluorothyazinone (**40**) inhibits the G-ve type III secretion system, GSK3882347 inhibits the binding of *E. coli* to host cell walls via FimH and ALS4 inhibits 4,4ʹ-diapophytoene desaturase (CrtN), which is an enzyme involved in the biosynthesis of staphyloxanthin. Finally, BVL-GSK098 (**58**) inactivates the TetR-like repressor, which reduces resistance to the TB drug ethionamide (**59**) and rescues its activity, which is conceptually similar to how BLIs restore the activity of BL antibiotics.

### Administration analysis

The administration routes (po, oral; IV/po, intravenous oral switch; IV, intravenous; IV/topical, IV and topical; po topical, CDI oral; topical; inhalation; n/d, not disclosed) of the small molecule antibacterial compounds under clinical development were analyzed by development phase (Fig. S1) and lead source (Fig. S1). Oral administration predominates and 19 of the 30 (~63%) are being developed against mycobacteria, which is pivotal as anti-TB drug combinations are taken for multiple months and are often administered in countries with limited capacity to deliver IV treatments. The second highest category is IV administration with 15, while there are four candidates that can be used both IV and po and two for both IV and topical. This IV/oral switch strategy is a competitive advantage as it can be implemented when patients move from hospital-based IV administration to oral administration in wards or at home. Four candidates are being trialed using the po topical administration route, which is used to treat gastrointestinal infections, such as *C. difficile* and *H. pylori*. For these infections, drugs are usually orally administered, but most are not significantly systemically absorbed, which reduces the potential for toxicity; however, one of the CDI clinical candidates, DNV-3837 (**25**) is being investigated using IV administration. There are four topically-only administered candidates, while murepavadin (**49**) is being trialed with inhaled administration to treat *P. aeruginosa* infections in the lungs of cystic fibrosis patients. This is being undertaken to more efficiently deliver **49** into the lungs, but it may also ameliorate kidney toxicity that was observed in a prior nosocomial pneumonia trial [273].

## Conclusion and outlook

The shape of the antibacterial pipeline has changed since our first analysis in 2011 [26]. At the front-end of the pipeline, there are now more than double the number of phase-I candidates (26) compared to 11 in 2015 [24] (Fig. 15). Funding initiatives have also helped to boost the number of phase-II (25) compounds since 2019 (18) (Fig. 15). Encouragingly, 16/26 (62%) of the compounds in phase-I and 14/25 (56%) in phase-II contain new pharmacophores (Figs. 16 and 17), with some also having new MoA (Table 8). Small molecule non-traditional antibacterial candidates are also starting to move through the pipeline with five in active development: fluorothyazinone (**40**), dovramilast (**42**), BVL-GSK098 (**58**), GSK3882347 and ALS4. Due to the increasing number of compounds with novel pharmacophores and targets in the pipeline (Table 8, Figs. 16 and 17), it is more likely that novel antibacterial drug classes will enter the clinic in the next few years, which is preferable to just expanding the pool of ‘me-too’ antibiotics. However, despite these early stage improvements, it is sobering to note that the overall antibacterial pipeline is still sparse compared to other therapeutic indications such as oncology (2,335 clinical trials in 2021 [371]) and even COVID-19 vaccines (180 in the pipeline in February 2023 [372]).

In contrast to the early-stage pipeline, the late-stage pipeline is still experiencing issues. There were only two new small molecule antibacterial drugs first approved between 2020 and 2022 (Table 1, Fig. 2): the fluoroquinolone levonadifloxacin (**1**) and its prodrug **2** in India in 2020 and the oxazolidinone contezolid acefosamil (**3**) in China in 2021. There was also one ‘non-traditional’ live biotherapeutic product, Rebyota, approved in the USA in 2022 (Table 2). The last first-in-class small molecule approval was the anti-TB diarylquinoline bedaquiline in 2012. However, this could change, if the current phase-III candidates, gepotidacin (**10**), zoliflodacin (**11**) and epetraborole (**13**) (Table 3, Fig. 4), all of which have new pharmacophores, were granted approval to treat gonorrhea and G-ve bacteria, gonorrhea, and *M. avium* complex (MAC) infections respectively. A future approval of the durlobactam (**60**) + sulbactam (**61**) combination for the treatment of *A. baumannii-calcoaceticus* (ABC) complex infections would also be a welcome addition to the antibacterial armamentarium. There has also been a steady but small decline in the number of phase-III candidates from 2013 to 2022 (Fig. 15). It will be critical to monitor how many of the phase-I and -II candidates, especially the compounds with new pharmacophores, move into Phase-III and beyond in the next few years.

In addition to the difficulty in identifying novel lead compounds suitable for antibacterial drug development, the ability to secure funding for phase-III trials and NDA/MAAs, as well as the capacity to generate adequate revenue to get positive net returns on investment for marketed antibacterial drugs [2–5], have been major obstacles to antibacterial drug development. Hopefully funding from organizations such as the AMR Action Fund [9, 10] will help to ameliorate some of these funding issues, while the successful implementation of pull initiatives should help to improve financial returns [11–16]. Another welcome addition has been the US FDA’s Limited Population Pathway for Antibacterial and Antifungal Drugs (LPAD) pathway that provides the potential for smaller, shorter, or fewer clinical trials (at least two phase-III trials are usually required) if the antibacterial drug candidate is “intended to treat a serious or life-threatening infection in a limited population of patients with unmet needs” [373]. However, the approved drug then carries a label restricting its use, which could limit future sales.

At least 19/26 (73%) phase-I compounds target G-ve bacteria (12 traditional compounds, one anti-virulence and six BL/BLI combinations), with four of these also possessing anti-G+ve activity. The high percentage of G-ve candidates being developed mirrors the clinical need and the recent focus of most funding schemes; however, the addition of G+ve activity to the 2022/23 CARB-X funding calls reflects the high mortality observed for global G+ve resistant infections in 2019 [1]. Only six of these 17 traditional G-ve antibacterial candidates are administered orally, with four of these being BL/BLI prodrugs. Although NPs have traditionally been the main source of novel antibacterials, 23 of the 34 (68%) of the compounds with new antibacterial pharmacophores were synthetically derived. There are also a substantial number of antitubercular drugs (TB and NTM) in the pipeline (19/62 (31%); one in phase-III, 12 in phase-II, and six in phase-I), showing the success of targeted funding for neglected diseases through organizations such as the TB Alliance (New York, NY, USA) and the Bill & Melinda Gates Foundation (Seattle, WA, USA).

In conclusion, despite the encouraging trends in phase-I and -II antibacterial drug candidates and plans to address issues with the late-stage pipeline, it is not the time to relax the urgency to continue to stimulate further antibacterial drug discovery and development.

## Supplementary information

Supplementary Information

## Footnotes

1 https://www.fda.gov/news-events/press-announcements/fda-approves-first-orally-administered-fecal-microbiota-product-prevention-recurrence-clostridioideshttps://ir.serestherapeutics.com/news-releases/news-release-details/seres-therapeutics-and-nestle-health-science-announce-fda

2 https://www.empr.com/home/news/drugs-in-the-pipeline/fda-panel-in-favor-of-sulbactam-durlobactam-for-acinetobacter-infections/
