# Correction: Antibiotics in the clinical pipeline as of December 2022

**DOI:** 10.1038/s41429-023-00671-6

**Published:** 2023-11-07

**Authors:** Mark S. Butler, Ian R. Henderson, Robert J. Capon, Mark A. T. Blaskovich

**Affiliations:** Centre for Superbug Solutions, Institute for Molecular Bioscience, The University of Queensland, St Lucia, Brisbane, QLD 4072 Australia

**Keywords:** Drug discovery, Microbiology

Correction to: *The Journal of Antibiotics*

10.1038/s41429-023-00629-8 published online 08 June 2023

The authors of the above article have noticed that prodrug contezolid acefosamil (**3**) was incorrectly stated as being approved rather than contezolid (**4**). This has been corrected throughout the review.

The changes are below:

In the sentence in the third paragraph of the introduction beginning “The new antibacterial drugs approved since the previous 2019 review…”, “contezolid acefosamil (**3**)” should have been “contezolid (**4**)”.

In Table 1, the drug name “contezolid acefosamil (**3**) (prodrug)” should have been “contezolid (**4**)”, without “(prodrug)”.

In the sentence beginning “Since the 2019 review…”, in paragraph 2 of the section “Antibacterial drugs launched from January 2013 to December 2022”, “contezolid acefosamil (**3**)” should have been “contezolid (**4**)”.

In the fourth paragraph of the section “Antibacterial drugs launched from January 2013 to December 2022”, several changes have been made. In the first sentence, “Contezolid acefosamil (**3**)” has been changed to “Contezolid (**4**)”, “(Youxitai, MRX-4, po)” has been changed to “(Youxitai, MRX-1, IV)”, and the word “prodrug” has been removed. In the third sentence, “(MRX-1)” has been removed, and “3” has been changed to “contezolid acefosamil (**3**)”. In the fourth sentence, “The prodrug” has been changed to “The prodrug **3**”.

In the “Conclusion and outlook” section, in the sentence beginning “There were only two new small molecule antibacterial drugs…”, “the oxazolidinone contezolid acefosamil (**3**)” should have read “the oxazolidinone contezolid (**4**)”.

In addition, there was also an error in the “New antibacterial pharmacophore analysis” section where it was stated that gepotidacin (**10**) inhibited GyrB and not GyrA. The mode of action of gepotidacin (**10**) is correctly described elsewhere in the review. GyrB has been corrected to GyrA.

The original article has been corrected.

