# Supplementary material for: Antibiotics in the clinical pipeline as of December 2022
Source: J Antibiot (Tokyo). 2023 Jun 8;76(8):431–73. doi: 10.1038/s41429-023-00629-8 (PMC10248350; doi:10.1038/s41429-023-00629-8)
Supplement: Supplementary file 1 — Supplementary Information [file 41429_2023_629_MOESM1_ESM.docx]

**Supplementary Information for ‘Antibiotics in the Clinical Pipeline as of December 2022’**

Mark S. Butler,* Ian R. Henderson, Robert J. Capon and Mark A.T. Blaskovich*

Centre for Superbug Solutions, Institute for Molecular Bioscience, The University of Queensland, St Lucia, Brisbane, 4072, Australia

Correspondence: Mark S. Butler, [m.butler5@uq.edu.au](mailto:m.butler5@uq.edu.au); Mark A. T. Blaskovich, [m.blaskovich@uq.edu.au](mailto:m.blaskovich@uq.edu.au)

**Overview of the drug development and approval process:**

Before clinical trials can start, an Investigational New Drug Application (IND) must be approved by the U. S. Food and Drug Administration (FDA), European Medicines Agency (EMA), Japanese Pharmaceuticals and Medical Devices Agency (PMDA), Indian Central Drugs Standard Control Organization (CDSCO), Chinese National Medical Products Administration (NMPA) or equivalent national agency. Upon successful completion of phase-III clinical trials, a New Drug Application (NDA: FDA, PMDA, CDSCO and NMPA) or a Marketing Authorization Application (MAA: EMA) must be approved to be able to market the drug. It is also possible to obtain approval for desperately required drugs under the FDA’s breakthrough or fast track approval programs after successful completion of phase-II trials. The FDA has also instituted a new Qualified Infectious Disease Product (QIDP) designation that allows for expedited review and an extra five years of market exclusivity for anti-infective drugs that target life-threatening infections.

**List of Clinical Trial Databases:**

Australia and New Zealand <http://www.anzctr.org.au/>

Clinical Trials Registry - India (CTRI) <http://ctri.nic.in/>

Europe <https://www.clinicaltrialsregister.eu/ctr-search/search>

Health Canada <http://www.hc-sc.gc.ca/dhp-mps/prodpharma/databasdonclin/index-eng.php>

Japan Primary Registries Network <https://www.umin.ac.jp/ctr/>

USA <http://clinicaltrials.gov>

Full list of Registries: <https://www.hhs.gov/ohrp/international/clinical-trial-registries/index.html>

**List of antibacterial clinical trial categories and pathogens:**

- *A. baumannii-calcoaceticus* (ABC)
- *C. difficile* infections (CDI) and *C. difficile*-associated diarrhea (CDAD)
- Carbapenem-resistant Enterobacteriaceae (CRE)
- Complicated intra-abdominal infections (cIAI)
- DAIR (debridement, antibiotics, and implant retention) surgical procedure
- Diabetic foot infections
- Early bactericidal activity (EBA), measures the ability of a single anti-TB agent to kill rapidly metabolizing mycobacteria present in Mtb pulmonary cavities
- Gonorrhea
- Non-tuberculous *Mycobacterium* (NTM)
- Periprosthetic joint infections (PJI)
- Pneumonia
  - Community-acquired bacterial pneumonia (CABP), previously community-acquired pneumonia (CAP)
  - Hospital-acquired bacterial pneumonia (HABP), previously hospital-acquired pneumonia (HAP)
  - Ventilator-associated bacterial pneumonia (VABP), previously ventilator-associated pneumonia (VAP)
- Pyelonephritis
- Skin and skin structure infections
  - Acute bacterial skin and skin structure infections (ABSSSI)
  - Complicated skin and skin structure infections (cSSSI)
  - Uncomplicated skin and skin structure infections (uSSSI)
  - Complicated skin and soft tissue infections (cSSTI)
- Tuberculosis (TB), *Mycobacterium tuberculosis* (Mtb)
  - Pulmonary *Mycobacterium avium* Complex (MAC)
- Urinary tract infections (UTI)
  - Complicated urinary tract infections (cUTI)
  - Uncomplicated urinary tract infections (uUTI)

**Other abbreviations:**

Antibody drug conjugate (ADC); β-lactam (BL); β-lactamase (BLI); Combating Antibiotic-Resistant Bacteria Biopharmaceutical Accelerator (CARB-X); diazabicyclooctane (DBO); Cryogenic electron microscopy (cryo-EM); extended-spectrum β-lactamases (ESBL); GlaxoSmithKline (GSK); Gram-positive (G+ve); Gram-negative (G-ve); intravenous (IV); natural product (NP); mode of action (MoA); monoclonal antibodies (mAbs); multi-drug resistant (MDR); novel bacterial topoisomerase inhibitor (NBTI); oral, *per orem* (po); penicillin binding protein, PBP; Pioneering Antimicrobial Subscriptions To End Up surging Resistance Act (PASTEUR Act); protein/mammalian peptide (P); synthetic (S); United States of America (USA); World Health Organization (WHO).

**Table S1** Antibiotics and β-lactamase inhibitor (BLI) combinations launched from 2000 to 2012, their antibacterial class, country of first approval and lead source

| ***Year*** | ***Drug name^a,b^*** | ***Class*** | ***Country of first approval*** | ***Lead source*** |
| --- | --- | --- | --- | --- |
| 2000 | linezolid | oxazolidinone | USA | S |
| 2001 | telithromycin | macrolide | Germany | NP-derived |
| 2002 | biapenem | carbapenem | Japan | NP-derived |
| 2002 | ertapenem | carbapenem | USA | NP-derived |
| 2002 | prulifloxacin | fluoroquinolone | Japan | S |
| 2002 | pazufloxacin | fluoroquinolone | Japan | S |
| 2002 | balofloxacin | fluoroquinolone | Republic of Korea | S |
| 2003 | daptomycin^b^ | lipopeptide | USA | NP |
| 2004 | gemifloxacin | fluoroquinolone | USA | S |
| 2005 | doripenem | carbapenem | Japan | NP-derived |
| 2005 | tigecycline | tetracycline | USA | NP-derived |
| 2007 | retapamulin^b,c^ | pleuromutilin | USA | NP-derived |
| 2007 | garenoxacin | quinolone | Japan | S |
| 2008 | ceftobiprole medocaril | cephalosporin | Canada | NP-derived |
| 2008 | sitafloxacin | fluoroquinolone | Japan | S |
| 2009 | tebipenem pivoxil | carbapenem | Japan | NP |
| 2009 | telavancin | glycopeptide | USA | NP |
| 2009 | antofloxacin | fluoroquinolone | China | S |
| 2009 | besifloxacinc | fluoroquinolone | USA | S |
| 2010 | ceftaroline fosamil | cephalosporin | USA | NP |
| 2011 | fidaxomicin^b^ | tiacumicin | USA | NP |
| 2012 | bedaquiline^b^ | diarylquinoline | USA | S |
| 2012 | perchlozone | thiosemicarbazone | Russia | S |

Abbreviations: G-ve, Gram negative; G+ve, Gram positive; NP, natural product; S, synthetic; USA, United States of America

^a^ The structures of the antibacterial drugs approved from 2000-2012 can be found in references [23–26].

^b^ First member of a new antibacterial drug or β-lactamase inhibitor (BLI) class approved for human therapeutic use.

^c^ Pleuromutilin derivatives have been previously used in animal health.

**Table S2** Miscellaneous list of other antibiotics and β-lactam (BL)/ β-lactamase inhibitor (BLI) combinations undergoing clinical development that do not fit within the review inclusion criteria

| **Name (Synonym)** | **Indication, Phase (Sponsor)** | **Comments** |
| --- | --- | --- |
| ATM-AVI (avibactam + aztreonam) (PF-0647387) | cIAI, HAP, VAP, phase-III (Pfizer) | New combination of approved antibiotic and BLI; NCT03329092 |
| tebipenem pivoxil (SPR994) | cUTI, phase-III (Spero Therapeutics) | Tebipenem pivoxil is approved as an antibiotic in Japan; NCT03788967 |
| WCK 4282 (cefepime + tazobactam) | cUTI, phase-III (Wockhardt) | cUTI, NCT03630081 |
| clofazimine (LAM320) | TB, phase-III (Wits Health Consortium)  Pulmonary *Mycobacterium avium* Complex and NTMs phase-II (various) | Clofazimine is a leprosy drug; NCT04062201, NCT02968212, and NCT05294146  Red Hill Biopharma is developing a combination of clarithromycin, rifabutin, and clofazimine for NTM, Phase-III (NCT04616924); also, other combinations in Phase-III and Phase-IV trials |
| fusidic acid (ACG-701, ARV-1801) | Melioidosis, CF, Phase-II (Idera Pharmaceuticals) | Fusidic acid is approved for use outside of the USA; NCT05105035 and NCT05641298 |
| ceftobiprole medocaril | ABSSSI and *S. aureus* bacteremia, phase-III (Basilea) | Phase-III trials completed in the US (ABSSSI - NCT03137173 and bacteremia -NCT03138733); approved for clinical use first in Canada in 2008 and later in other countries |
| gallium nitrate (Ganite) | NTMs / Cystic Fibrosis, phase-II (Cystic Fibrosis Foundation) | NCT02354859. Disrupts bacterial iron metabolism through iron substitution; Ganite is used as a treatment for symptomatic hypercalcemia secondary to cancer |
| HY-001 (colistin methanosulphonate and zidovudine) | UTI, phase-II (Helperby Therapeutics) | Combination of antiviral HIV-1 drug zidovudine and colistin; EUCTR2019-000910-12-PL  Described in *Int. J. Antimicrob Agents*. **2019** 54:55−61, doi: [10.1016/j.ijantimicag.2019.04.011](https://doi.org/10.1016/j.ijantimicag.2019.04.011) |
| teicoplanin (inhalation) | MRSA cystic fibrosis, phase-I (Neupharma and Italfarmaco) | Teicoplanin is used outside of the USA as an antibiotic; NCT04176328 |

**Figure S1** Comparison of the administration routes of antibacterials in clinical development (po, oral; IV/po, intravenous and oral switch; IV, intravenous; IV/topical, intravenous and topical; topical, topical, spray or drops; po topical, CDI oral; inhalation; n/d, not disclosed) by development phase

**Figure S2** Administration routes (po, oral; IV/po, intravenous and oral switch; IV, intravenous; IV/topical, intravenous and topical; topical, topical, spray or drops; po topical, CDI oral; inhalation; n/d, not disclosed) of antibacterials in clinical development by small molecule lead source (NP, NP-BLI, P, S and S-BLI)
